# Supplementary material for: Molecular evolution and expression divergence of three key Met biosynthetic genes in plants: CGS, HMT and MMT
Source: PeerJ. 2018 Dec 4;6:e6023. doi: 10.7717/peerj.6023 (PMC6284425; doi:10.7717/peerj.6023)
Supplement: Supplemental Information 1 [file peerj-06-6023-s001.rtf]

AthCGS1    1 ------------------------------MAVSSFQCPTIFSSSSISGFQCRSDPDLVGSPVGGSS----RRRVHASAG
AthCGS2    1 --------------------------------------------------------------------------------
BraCGS1    1 ------------------------------MAVLSFQSPTNFSP----------------TSIPGSS----RRRFDSTAG
BraCGS2    1 --------------------------------------------------------------------------------
BraCGS3    1 ------------------------------MAVSSFQCPTIFSIP---SFQCRSDPDLVGSPIGGSS----RRRFNPTAG
GmaCGS1    1 ------------------------------MAVSSSHMR--------FTFECRSDPDFSPPPPSFDN----LRRRNFRSS
GmaCGS2    1 ------------------------------MAVSSSHMR--------FTFECRSDPDFSPPPPSFDN----LRRRNFRSS
MdoCG1     1 ------------------------------MAV--CTYPPRVFAS--------SQP-------GARP-----RLSGRVDP
MdoCGS2    1 ------------------------------MAV--CSYPPRVFTS--------SQP-------GTRP-----RLSGRVDP
MdoCGS3    1 ------------------------------MAV--CTYPPRVFAS--------SQP-------GARP-----RLSGRVDP
MtrCGS1    1 ------------------------------MAVSSFHRV--------FTFECRSDPDFTSLPSTDNH----HHNRRRHFP
MtrCGS2    1 ------------------------------------MNA--------FDISARS---------------------SCHNI
SlyCGS     1 ------------------------------MAVSSYARAFPSFECR-------SEPDFSGSLPHPKA---GVRFSGKYNS
VviCGS     1 ------------------------------MAVSSCPRVSAAFECR-------SEPDFCGGPHRPEAFASGGRFSGKASS
PvuCGS     1 ------------------------------MAVSSSHMR--------FTFECRSDPDFSPPP-SFDN----LRRRNFR-S
TcaCGS     1 ------------------------------MAVSSCSCPPRVFNAY-SSFECRSDPDFSGAPIGDKP-----RVRPSRRL
BolCGS1    1 ------------------------------MAVSSFQCPTIFSIP---SFQCRSDPDLVGSPIGGSS----RRRFNATAG
BolCGS2    1 ------------------------------------------------------------MALYRSI----SLLSVSSDP
AcoCGS     1 ------------------------------MAVAGGFARVLPS-----SFECRSDPDFSTGSEKFNSTG--NRFTGKTNS
OsaCGS1    1 --------------------------------------------------------------------------------
OsaCGS2    1 --------------------------------------------------------------------------------
OsaCGS3    1 --------------------------------------------------------------------------------
OsaCGS4    1 --------------------------------------------------------------------------------
OsaCGS5    1 --------------------------------------------------------------MATVS---SLPSPAFLAA
ZmaCGS1    1 --------------------------------------------------------------------------------
ZmaCGS2    1 --------------------------------------------------------------------------------
ZmaCGS3    1 --------------------------------------------------------------------------------
ZmaCGS4    1 --------------------------------------------------------------MATVS---LTP-QAVFST
SitCGS1    1 MQDALTNQTHHSTTNKPHRISKGLTSATQSPTGSRRNRSSRSPLPARPPPRHQRNPSSASSAMATVS---LTP-QVVFST
SitCGS2    1 --------------------------------------------------------------------------------
SitCGS3    1 --------------------------------------------------------------------------------
BdiCGS1    1 --------------------------------------------------------------------------------
BdiCGS2    1 --------------------------------------------------------------MATLS---FSS-PPSFAG
SbiCGS1    1 --------------------------------------------------------------------------------
SbiCGS2    1 ------------------------------------MKSKVERRHEHEGITNVTNENQLDELLPPPT---PWP-PSRSLR
MacCGS1    1 -------------------------------------MAVSSTIY----PAYFVSAAAGDRRTSDSG---AHPRPEKLGS
MacCGS2    1 -------------------------------------MAISSALCLPSRPHSKPYFSAADCRSSDSG---ARPRIEMPAA
ZomCGS     1 --MYIYRANGHYPSSRLCTLHHIHIPRVESRFFDMAFITSTFQQTPSSSLPFVPRAVAVDRVFSFTT---GRERSNFFSS
AtrCGS     1 ------------------------------MAVSSCSILGGCPRIFP-SFECRSDPDFTGHFQSEKQ--------KTRGF
SmoCGS     1 --------------------------------------------------------------------------------
SfaCGS1    1 ----------------------MATVCILQAGAAATSMLPGVGVGVGG---CHDCRPDASGASSTKTNAPPSSGGVGGGG
SfaCGS2    1 ----------------------MAAVCILQAGVATSTLGAGGGGGGGGGGGHHDCRPDTSGASSSKTAGTSSSG------
MpoCGS     1 ---------------------MAAMSSTLRAGGPMGYGVAFDCRPDPAGTSSRASAQKPFCRASCGDDSAPGKS------
PpaCGS1    1 ---------------------MAAALCIGQAAAS--------AMPSAG----YEYRPDASGGAASGKSGFGGSE------
PpaCGS2    1 ---------------------MAAALCIGQAAAAAAVAAS--AMPSVG----YEHRPDASGGAASGKSGLGGGD------
CreCGS     1 --------------------------------------------------------------------------------
VcaCGS     1 --------------------------------------------------------------------------------
PglCGS     1 -----------------------------MAVANSCMMMGAQIAGQARAPPLFECRADTSGSAKHERPDSGSSP------
PsiCGS     1 -----------------------------MAVANSCMMMGAQIAGQARAPPLFECRADTSGSAKHERPDSGSSP------


AthCGS1   47 ISSSFTGDAG----LSSRILRFPPNFVRQLSIKA-RRNCSNIGVAQIVAAKWSNNPSSALPSAAAAAATSSASAVSSAAS
AthCGS2    1 --------------------------------------------------------------------------------
BraCGS1   31 ISSSFTGDGP----ISSMILRFPPNFVRQLSIKA-RRNCSNIGVAQIVAAKWSNNPSSGLPSAAAAASS-----------
BraCGS2    1 --------------------------------------------------------------------------------
BraCGS3   44 MPSPFTGGGR----MSSMILRFPPNFVRQLSIKA-RRNCSNIGVAQIVAAKWSDSGSP--PAVAAAASS-----------
GmaCGS1   39 AGSGAAFHG-----ISSLILRFPPNFQRQLSTKA-RRNCSNIGVAQIVAASWSNNSDNSPAAGAPAPPA-----------
GmaCGS2   39 AGSGAAFHG-----ISSLILRFPPNFQRQLSTKA-RRNCSNIGVAQIVAASWSNNSDNSPAAGAPAPPA-----------
MdoCG1    29 TASVYR--------LSSPILRFPPNFVRQLSTKA-RRNCSNIGVAQIVAASWSNNTPNSGVPAAPSATA-----------
MdoCGS2   29 TVPVHG--------LSSLILRFPPNFVRQLSTKA-RRNCSNIGVAQIVAASWSNNNPNSGVSAAPSATA-----------
MdoCGS3   29 TASVYR--------LSSPILRFPPNFVRQLSTKA-RRNCSNIGVAQIVAASWSNNTPNSGVPAAPSATA-----------
MtrCGS1   39 TTSLSSAAA-----ISSPILRFPPNFQRQLSTKA-RRNCSNIGVAQIVAASWSNEGTGNPNAGVPTPVP-----------
MtrCGS2   16 ALATTPFRH-----LSSSYHLMTLKLQHHVSIGE-RR---SIRVVRITASLWPN---TDSTVSQPSP-------------
SlyCGS    41 GSNRSQVYG-----LSSLIYRFPPNFVRQLSIKA-RRNCSNIGVAQVVAASWSNNQAGPEFTPAANAVD-----------
VviCGS    44 VPS-HQIFGASA--LSSLIFRFPPNFVRQLSTKA-RRNCSNIGVAQVVAASCD---------------------------
PvuCGS    37 VGSGASFHG-----VSSLILRFPPNFQRQLSTKA-RRNCSNIGVAQIVAASWSNNSTNNPSAGAPAPPA-----------
TcaCGS    45 TAASFCTGGIGGGGLSSLIFRFPPNFVRQLSIKA-RRNCSNIGVAQIVAASWSNSPASGSPSSAAAAAQ-----------
BolCGS1   44 MSSPFTCGGR----MSSTILRFPPNFVRQLSIKA-RRNCSNIGVAQIVAAKWSNN--------PASGLP-----------
BolCGS2   17 QPSCFTGYGR----VSSFILRFPPNIARQLSTKA-RGNCNNFSVAQTVVAKWSNSTGSGLTSVPAVTSV-----------
AcoCGS    44 FPICGSFNSG---LSSSLIFRFPPNFVRQLSIKA-RRNCSNIGVAQVVAASWSNNNNGNSVPNVSAVDS-----------
OsaCGS1    1 -------------------MAKSSSLVT-KVLVQRRRRVHRSRRSP-TKPER----------------------------
OsaCGS2    1 -------------------MAKSSSLVT-KVSAQRRRRVHRPRRSP-TKPERSAAAVALSHEKILLA-------------
OsaCGS3    1 -------------------MTLRRRSGAGGTPPTTRSSS-----------------------------------------
OsaCGS4    1 -------------------MASSVALVLMKASLPRRQRMDRPRRSP-AAVQLVAG-------------------------
OsaCGS5   16 DPAAA--------LPSATILRFPPNFVRQLSTKA-RRNCSNIGVAQIATA------------------------------
ZmaCGS1    1 --------------------------------------------------------------------------------
ZmaCGS2    1 --------------MTSSVMAPSSKLVKTTSLPSHQRSLVQPLRSP--RHKRISAMRRAL--------------------
ZmaCGS3    1 -------------------MAPSSKLVKTNSLPSHQRSLVQPLGSP--RHKRVSALFRAP--------------------
ZmaCGS4   15 ESGGA--------LASATILRFPPNFVRQLSTKA-RRNCSNIGVAQIVAAAWSDCPAARPHLGGGG--------------
SitCGS1   77 ESGGA--------LSSATILRFPPNFVRQLSTKA-RRNCSNIGVAQIVAAAWSDRPAARPNSGGGGGGRAR---------
SitCGS2    1 -------------------MAPSTSLGKIS-LPRQQRRLLQPIHSP--RHKRVTAAHPA---------------------
SitCGS3    1 -------------------MAPSTSLGKIS-LPRQQRRLLQPIHSP--RHKRVTAAHPA---------------------
BdiCGS1    1 -------------------MARSSSAVK--SSLPKQRRSLRPRRSPKPNFQRHAAALN------PDA-------------
BdiCGS2   15 DNSGA--------LTSATILRFPPNFVRQLSTKA-RRNCSNIGVAQIVAAAWSDRSARPSHTGGGCRARG----------
SbiCGS1    1 -------------------MAPSSKLVKTSSLPKHQRSLVQPLHSP--RHKRVSALCPAA--------------------
SbiCGS2   41 RRSSP--------PSPAAPWPLPPSSASRQTSSA-SSAPRHAATAATSASRRSSPPRGQTAPPLAP--------------
MacCGS1   37 SSFLLGGAPRFSGLSSATILRFPPNFVRQLSTKA-RRNCSNIGVAQIVAASWSNSSQSFEAP------------------
MacCGS2   41 SSLRG-------GRASPLILRFPPNFVRQLSIKA-RRNCCNIGVAQIAAASWSNDPPAFEGP------------------
ZomCGS    76 KEIGPNPNCQV-ISPGSKILRFPSNFVRQLSNKA-RRNCSNIGVAQVVAASWSDGNRESSFSASAYGDSAL---------
AtrCGS    42 SGQIHGFSG------QSLILRFPPNFVRQLSIKA-RRNCSNIGVAQVVAASWSNGPIASEEKAIGVVAP-----------
SmoCGS     1 -----------------MILRFPPNFARQLSVKS-RRNCSNIGVAQVVAASAMSGQSSGGSSSIVDGVAAALS-------
SfaCGS1   56 KRSSGFDLSSNGFLRPPRIPRLPPNIVRQLSIKA-RRNCSNIGVAQVVAASATDRPSLDAAISTLPQSADVLK-------
SfaCGS2   53 VKTSGFDLS-NGFLRPPVIPRLPPNIVRQLSIKA-RRNCSNIGVAQVVAASATDRPSLD-AISALPQ-ADVLE-------
MpoCGS    54 STQSSKLNRSLACDSPPFILRFPPNFVRQLSIKA-RRNCGNIGVAQVVAASATDLPAVSSENVVAPASADLEK-------
PpaCGS1   42 --QVPVNVARKGFFCPPCITRFPPNFVRQSSIKA-RRNCSNIGVAQVVAASATDRSNPAAVAAA-LTSSTQAE-------
PpaCGS2   48 --KTPLNIASKGFFCPPCITRFPPNFVRQSSIKA-RRNCSNIGVAQVVAASATDRSNPAAVSAAGLSSSAHAE-------
CreCGS     1 ----------MR-SVYSPAAEMPAGVSMRAPVPAALRSAARSGLHLRNASFHTHRPS-----VVAPAAAKDLE-------
VcaCGS     1 -------MFGARGLCHVSAADMPATVSMRAPIPASLRPAARSGLHLRQAAFHRRRPSSI--GVICGAAAKDLE-------
PglCGS    46 -PTGFLKRGACFGASAPLILRFPPNFVRQLSIKA-RRNCSNIGVAQVAAASVTNIPVSS-------APANAGE-------
PsiCGS    46 -PTGFLKRGACFGASAPLILRFPPNFVRQLSIKA-RRNCSNIGVAQVAAASVTNIPVSS-------APANAGE-------


AthCGS1  122 AAAASSAAAAPVAAAPP-VVLKSVDEEVVVAEEG----IREKIGSVQLTDSKHSFLSSDGSLTVHA--------------
AthCGS2    1 -----MQILKENASNQRFVTRESEVN------------------RICCGFN--GFQLG----------------------
BraCGS1   95 ---VSAAASAPAAAVPPVVALNGVDEEVVAAEG------AREIG--LKKDSKPSFLSSDGSLTVHA--------------
BraCGS2    1 --------------------------------------------------------------------------------
BraCGS3  106 ---ASAAASAPAAAVPP-VALNGADEEVVAAEG------IREIGSVQLKDSKPSFLSSDGSLAVHAVFVGVYVMMI----
GmaCGS1  102 ------ATAADAATVPLPVDLAAAEDVVVS------AAAAENGTVQLNSSSYS-FLKSDASKTIHA--------------
GmaCGS2  102 ------ATATDAATVPLPVVVAANEDVVVS------AAADENGAVQLNSSSYSSFLKSDASKTIHA--------------
MdoCG1    89 -----VDAAATSAIP------VDPAQISGGDEVAVFGNG-VQLGEALPDLKEASFLSSDGSLAIHA--------------
MdoCGS2   89 -----VDAAATAAIS------VDPAQISGGDEVAVFENG-VQLGEALP-LKEASFLSSDGSLAIHA--------------
MdoCGS3   89 -----VDAAATSAIP------VDPAQISGGDEVAVFGNG-VQLGEALPDLKEASFLSSDGSLAIHA--------------
MtrCGS1  102 ------AVASAVDATTAPVPLDLDADVTDG-------DVVENGAVQTNRSSYSSFLKSDASKTIHA--------------
MtrCGS2   71 --------------------------------------AESNGALEID-SPYS-FLKCDGSKTVHA--------------
SlyCGS   104 -----SSASAAVTSIGITTGDEEVAVVENAD----CSDQNVQIKGSTG-VKYASFLNSDGSVAIHA--------------
VviCGS    93 --------------------------VQYED----LAD------------EKASFLGFDGTLTIHA--------------
PvuCGS   100 ------VSAADAATAPLLVNISADEDIVVSTNDAVASAADNNGVVQLNRSSYSSFLKSDASKTIHA--------------
TcaCGS   113 -----TATAATVHVS------DDVAFVQGCN-----DNGSVQIGGSDN--STTSFLSSDGSIAVHA--------------
BolCGS1  100 ---PAAASPGAAPAVPP-VALNGVDEEVAAAEG------IREIGSVQLKDSKPSFLSSDGSLAVHA--------------
BolCGS2   81 ----DEEVLVAAESVKEMVSIKSEDVTHMKPSFL----KSDGSLTVHAGTPPLEYPSS----------------------
AcoCGS   109 ---AAAATEIEIDEVKAVVDVNGDNSKINNN------NNNNGVQLSGLAALKASFLCSDGSIAVHA--------------
OsaCGS1   32 -------AAAADTFGSLAGCPSSP-EYPVVPDLDDCVVDTAVDDAPVVAAEAAGRRASDETLAVHAG-------------
OsaCGS2   47 -----GVDAAADMFGSLAGCPSSP-EYPVVPDMDDRYVDGAVDDAP---AAAAGRRASDETLAVHAG-------------
OsaCGS3   21 ----------------------------------------------------TPGSGGARAIGGVAA-------------
OsaCGS4   36 ------VDAADDMFGSLAGCPPRPLEYPVVPDLDDYDVD----DAP---VAGRPAPASDKTLAVHAG-------------
OsaCGS5   57 ----------------------EVGAIPNAK-------LGQPSAAALAEQ---ALLGSDASLAVHAGNHDQSPLRSIAFP
ZmaCGS1    1 --------------------------------------------------------------------------------
ZmaCGS2   45 -----ESSMPQEELVTAAAGDADH--------------------------QPRARRASDETLAVHAG-------------
ZmaCGS3   40 -----ESSLLQEEFVTAASGEAHH--------------------------QARARRASDETLAVHAG-------------
ZmaCGS4   72 ----------------------EVSAIPNAK-------VAQPSAVVLAER---NLLGSDASLAVHAG-------------
SitCGS1  139 -GVASSHAAAASAAAVAASAAAEVSAIPNAK-------VAQPSAVVQAER---KLLGSDASLAVHAG-------------
SitCGS2   38 -------ALLQELFGDAEP--------------------------------PKPRRASDETLAVHAG-------------
SitCGS3   38 -------ALLQELFGDAEP--------------------------------PKPRRASDETLAVHAG-------------
BdiCGS1   41 -----LVDATLPVFEPEANHPGCADQDMVVPT-----------------AARRRRSVSDETLAVHAG-------------
BdiCGS2   76 ----ATSHAAAASAAASATAAAEVGAIPNAK-------LAQPSAAALAER---TLLGSDASLSVHAG-------------
SbiCGS1   40 -----PESLLQELVTAAAACDAHH--------------------------QPRAKRASDETLAVHAG-------------
SbiCGS2   98 -----------------TQAAAEVSAVPNAK-------VAQPSAVVLAER---NLLGSDASLSVHAG-------------
MacCGS1   98 ----------------------RSAVSADAV-------VAPEVSAAVAKA--AASLSSDGSLAVHAG-------------
MacCGS2   95 ----------------------RPAASSDAS-------ADAKAS-VVAKT--AALFSSDGSLAVHAG-------------
ZomCGS   145 -SAVPEVEAIPSVLKDGDGVASGESTTDESG-------VLQSLGLIEDSRPPFPIFRFDGSLTVHAG-------------
AtrCGS   104 ------VIDSSCNNN------------------------NTEVVQSEFTDSNDSFLSSDGSLAVHA--------------
SmoCGS    56 ---VGISVNGTAVKIDKA--------------------------------AEKPKPKSTSTLAVHG--------------
SfaCGS1  128 ---AQQQLGSSAHATDGGAAATSTFVIDSTLLSQAIAATTTSSCNNGSLPVEKSKPMTRGTLAVHG--------------
SfaCGS2  122 ---GQQ-LG-SAHATAG----ASSFSIDTTLLSQAIAAP-----RNGTLPVEKAKPMARATLAVHG--------------
MpoCGS   126 ---PLFEAGAALSSTAVVNG------------------------VNSSATRLSEKPRSCSTTSVHG--------------
PpaCGS1  111 ---LAN-IGNGSAVVSAD---ANEALLES----GTSLAS------AGASETEKPRRSKSSTLAVHG--------------
PpaCGS2  118 ---VVDELGNGSAAAPTG---ANEALLDSLIASDDTLSR------EKSSEAKKSSRSKPSTLAVHG--------------
CreCGS    58 ---PTSASSNGAPKNNG------------------------------------VQFTKLSTTAVHG--------------
VcaCGS    65 ---ATSASSNGTSLQHE------------------------------------VKPSKLSTAAVHG--------------
PglCGS   110 ---PFVLTCNNSVQADG--------------------------------ALVEDFVSPDGTLAVHS--------------
PsiCGS   110 ---PFVLTCNNSVQADG--------------------------------ALVEDFVSPDGTLAVHS--------------


AthCGS1  183 ------------------GERLGR-----------GIVTDAITTPVVNTSAYFFKKTAELIDFKEK------RSVSFEYG
AthCGS2   34 -------------------ERLRR-----------DMKTSSITTLVVNTTTYFFKDTTELIDFKEK------RIDLYEYA
BraCGS1  150 ------------------GERLGR-----------GIVTDAITTPVVNTSAYFFNKTADLIDFKEK------RSVSFEYG
BraCGS2    1 --------------------------------------------------------------------------------
BraCGS3  172 ------------------GERLGR-----------GIVTDAITTPVVNTSAYHFKNTAELLDFKEK------RSVSFEYG
GmaCGS1  155 ------------------AERLGR-----------GIETDGITTPVLNTSAYFFKKTADLIDFKEN------RQVSYEYG
GmaCGS2  156 ------------------AERLGR-----------GIETDGITTPVVNTSAYFFKKTADLIDFKEN------RQVSYEYG
MdoCG1   143 ------------------GERLGR-----------GIVTDAITTPVVNTSAYFFKKTADLIDFKEK------RATSFEYG
MdoCGS2  142 ------------------GERLGR-----------GIVTDAITTPVVNTSAYFFKKTADLIDFKEK------RATSFEYG
MdoCGS3  143 ------------------GERLGR-----------GIVTDAITTPVVNTSAYFFKKTADLIDFKEK------RATSFEYG
MtrCGS1  155 ------------------AERLGR-----------AIVTDGITTPVVNTSAYFFKKTADLIDFKEK------RQTSYEYG
MtrCGS2   97 ------------------AERLGR-----------GIVTDAITTPVVNTSVYFFKKTADLIDWKEK------RQFSYEYG
SlyCGS   160 ------------------GERLGR-----------GIVTDAITTPVVNTSAYFFNKTSDLIDFKEK------RRASFEYG
VviCGS   117 ------------------GERLGR-----------GIVTDAITTPVVNTSAYFFKKTAELIDFKEK------RRASFEYG
PvuCGS   160 ------------------AERLGR-----------GIVTDGITTPVVNTSAYFFKKTADLIDFKEN------RQVSFEYG
TcaCGS   161 ------------------GERLGR-----------GIVTDAITTPVVNTSAYFFKKTQELIDFKEK------RHKSFEYG
BolCGS1  156 ------------------GERLGR-----------GIVTDAITTPVVNTSAYHFKNTAELLDFKEK------RSVSFEYG
BolCGS2  131 -------------------ERLDR-----------GIVKDAITTPVVNSSAFVFKKTADVLDFKEK------RVVRHEYG
AcoCGS   166 ------------------GERLGR-----------GIVTDGITTPVVNTTAYWFKNSDELIDFKEG------RYASFEYG
OsaCGS1   91 -------------------EKLG----SG------AAETDSIATPIVSGTTHWFRDSADLIAFREG------WRRSFEYG
OsaCGS2  105 -------------------EKLG----SG------AAETDSIATPIVSGTTHWFRDSADLIAFREG------RRRSFEYG
OsaCGS3   36 -------------------EVTD----------------YSIATPIVTGTARWFRDSADLVAFREGGGRRRRRRHSFEYA
OsaCGS4   90 -------------------EKLG----SGAA----AAETDSIATPIVGGTTHWFRDSADLIAFREG------RRRSFEYG
OsaCGS5  105 PLLRFFSDPFRAFDIPIEGERLGR-----------RIATDAITTPVVNTSAYWFNNSQELIDFKEG------RHASFEYG
ZmaCGS1    1 --------------------------------------------------------------------------------
ZmaCGS2   81 -------------------EKLG-KKDAGVGGSGTGIETDSMATPIVSGTTHWFKSSEDLIAFKEG------RRHSFEYG
ZmaCGS3   76 -------------------EKLAGKKDADVGGTGT--ETDSTATPIVSGTTHWFKSSEDLIAFKEG------RRHSFEYG
ZmaCGS4  107 -------------------ERLGR-----------RIATDAITTPVVNTSAYWFNNSQELIDFKEG------RHASFEYG
SitCGS1  195 -------------------ERLGR-----------RIATDAITTPVVNTSAYWFNNSQELIDFKEG------RHASFEYG
SitCGS2   66 -------------------EKLG--KGADEA------ATDSIATPIVSGTTHWFKSSEDLIAFKEG------RRHSHEYG
SitCGS3   66 -------------------EKLG--KGADEA------ATDSIATPIVSGTTHWFKSSEDLIAFKEG------RRHSHEYG
BdiCGS1   86 -------------------EKLG----KDG-----MAETDSIATPVVSGTTHWFRSSEDLIAFKEG------RRHSFEYG
BdiCGS2  129 -------------------ERLGR-----------RIATDAITTPVVNTSAYWFSSSQELIDFKEG------RHASFEYG
SbiCGS1   76 -------------------ENLG-KKGADVVGTGTGTETDSIATPIVSGTTHWFKSSEDLIAFKEG------RRHSFEYG
SbiCGS2  138 -------------------ERLGR-----------RIATDAITTPVVNTSAYWFNNSQELIDFKEG------RHASFEYG
MacCGS1  134 -------------------ERFGR-----------GITTDAITTPVVNTSAYWFNNSDELIDFKEK------RHASFEYG
MacCGS2  130 -------------------ERFGR-----------GISTDGITTPVVNTSAYWFSNSDELIDFKEK------RHASFEYG
ZomCGS   204 -------------------ERSGR-----------GIVTDGITTPIVNTSAYWFKNTAELIDFKEG------RHDSFEYG
AtrCGS   140 ------------------GERIGR-----------GIVTDAITTPVVNTSAYLFKTTAQLIDFKEG------RFASFEYG
SmoCGS    87 ------------------GERENG-----------TKVADSLTTPIVQTATYTFRNTADLIAFQAR------ENN-FEYG
SfaCGS1  191 ------------------GERTGR-----------RDVRDALITPICQTSTYFFKDTAELIAFQEG------THTSFEYG
SfaCGS2  174 ------------------GERTGH-----------RNVQDALITPICQTSTYFFRDTAELIAFQEG------THTSFEYG
MpoCGS   165 ------------------GERNGR-----------PKVSDALTTPIVQTSTYFFRNTAELISFQEG------SHTSFEYG
PpaCGS1  160 ------------------GERTRR-----------PKVQDTLTIPICQTSTYTFKDTAELIAFQEG------TFTSFEYG
PpaCGS2  172 ------------------GERTRR-----------PKVQDTLTIPICQTSTYTFKDTAELIAFQEG------TFTSFEYG
CreCGS    85 ------------------GERGGR-----------PRVADALTTPIVQTSTYHFRNTAELIEYNEG------RFDSYEYG
VcaCGS    92 ------------------GERAGR-----------PRVADALTTPIVQTSTYHFRNTAELIEYNEG------RFDSFEYG
PglCGS   141 ------------------GEKTGR-----------AIVTDAIATPIVQTSTYTFKNTAELIAFQEG------RHISYEYG
PsiCGS   141 ------------------GEKTGR-----------AIVTDAIATPIVQTSTYTFKNTAELIAFQEG------RHISYEYG

AthCGS1  228 RYGNPTTVVLEDKISA----------LEGAESTLVMASGMCASTVMLLALVPAGGHIVTTTDCYRKTRIFMENFL-PKLG
AthCGS2   78 RYGNPTTMALEEKISV----------LEGAESTLVMASGMYASNVMLLALVPTNGHIVATKDCYKETRIFMENFL-TKLG
BraCGS1  195 RYGNPTTIVLEEKISA----------LEGAESTLVMASGMCASTVMLLALVPAGGHIVTTTDCYRKTRIFMENFL-PKMG
BraCGS2    1 -----------------------------------MASGMCASTVVLLAMVPRGGHIVTTTDCYKETRMFIETFL-PKLG
BraCGS3  217 RYGNPTTIVLEDKIS-----------------------------------------------------------------
GmaCGS1  200 RYGNPTTVVLEEKISI----------LEGAESTVIMASGMCASVVLFMALVPAGGHLVTTTDCYRKTRIFIETFL-PKMG
GmaCGS2  201 RYGNPTTVVLEEKISA----------LEGAESTVIMASGMCASVVLFMALVPAGGHLVTTTDCYRKTRIFIETFL-PKMG
MdoCG1   188 RYGNPTTVVVEEKI----------SALEGAESTMILASGMCASTVMLMALVPAGGHIVTTTDCYRKTRIFIETIL-PKMG
MdoCGS2  187 RYGNPTTVVVEEKIRPCCGELVMVSALEGAESTMILASGMCACTVMLMALVPAGGHIVTTTDCYRKTRIFIETIL-PKMG
MdoCGS3  188 RYGNPTTVVVEEKI----------SALEGAESTMILASGMCASTVMLMALVPAGGHIVTTTDCYRKTRIFIETIL-PKMG
MtrCGS1  200 RYGNPTSTVLEEKISE----------LEGAESTILMASGMCASIVLLMALVPAGGHLVTTTDCYRKTRIFIETVL-PKMG
MtrCGS2  142 RYGNPTTAVLEEKMSA----------LEGAESTLFLASGMCTSSVMFLALVPAGGHIVTTTDCYRRTRIFIANML-PKMG
SlyCGS   205 RYGNPTTVVLEEKI----------SALEGAESTLIVASGMCASTVMFLALVPAGGHIVTTTDCYRKTRVFIETIL-PKMG
VviCGS   162 RYGNPTTVVLEEKI----------SALEGAESTVIMASGMCASTVMLLALVPPGGHMVTTTDCYRRTRIFIETFL-PKLG
PvuCGS   205 RYGNPTTVVLEEKISE----------LEGAESTVIMASGMCASVVLFMALIPAGGHLVTTTDCYRKTRIFIETFL-PKMG
TcaCGS   206 RYGNPTTVVAEEKI----------SALEGAESTLIVASGMCVSTVMLMTLVPAGGHIVTTTDCYRKTRIFIETIL-PKMG
BolCGS1  201 RYGNPTTIVLEDKISA----------LEGAESTLVMASGMCASTVMILALVPAGGHIVTTTDCYRKTRIFMETFL-PKMG
BolCGS2  175 RYGNPTTLVLEKKISA----------LEGAESTLVMASGMCASTVMLLALVPRNGHVVTTTDCYRKTRIFMETFL-PKLG
AcoCGS   211 RYGNPTTESLERKISA----------LERAETTLFLASGMCASTVLMFALVKKGGHIVTTTDCYRKTRIFIQDYLVPNMD
OsaCGS1  136 RYGNPTVKVLEEKISA----------LERAEATLVTSSGMNAIVATLLALVPPGGHVVVTADCYSEARAFIHDKL-SKMG
OsaCGS2  150 RYGNPTVKVLEEKISA----------LERAEATLVTSSGMNAIVATLLALVPPGGHVVATADCYSEARAFIRDKL-SNMG
OsaCGS3   81 RYGNPTVEVLEDKISA----------MEKAEATIVTSSGMNAIVATLLAVVPPGGHVVATTDCYSEARAFIRDRL-SKMG
OsaCGS4  137 RYGNPTVKVLEEKISA----------LERAEATLVTSSGMNAIVATLLALVPPGGHVVATADCYSEARAFIRDKL-SNMG
OsaCGS5  168 RYGNPTTEALEKKMSA----------LEKAESTVFVASGMYASVAMLSALVPAGGHVVTTTDCYRKTRIYMETEL-PKRG
ZmaCGS1    1 -------------MSA----------LEKAESTVFVASGMYAAVAMLSALVPAGGHIVTTTDCYRKTRIYMENEL-PKRG
ZmaCGS2  135 RYSNPTVKVLEEKISA----------LERAEATLVTSSGMNAITTTLLALVPPGGHVVTTTDCYGEARAFIRDRL-SVMG
ZmaCGS3  129 RYSNPTVKVLEEKISA----------LERAEATLVTSSGMNAITTTLLALVPPGGHIVTTTDCYSEARVFIRDRL-SMMG
ZmaCGS4  151 RYGNPTTEALEKKMSA----------LEKAESTVFVASGMYAAVAMLSALVPAGGHIVTTTDCYRKTRIYMETEL-PKRG
SitCGS1  239 RYGNPTTEALEKKMSA----------LEKAESTVFVASGMYAAVAMLSALVPAGGHIVTTTDCYRKTRIYMENEL-PKRG
SitCGS2  113 RYSNPTVKVLEDKISA----------LERAEATLVTSSGMNAIVATLLALVPPGGHVVTTTDCYSEARAFIRDRL-SKMG
SitCGS3  113 RYSNPTVKVLEDKISA----------LERAEATLVTSSGMNAIVATLLALVPPGGHVVTTTDCYSEARAFIRDRL-SKMG
BdiCGS1  132 RYGNPTVKVLEEKISA----------LERAEATLVTSSGMNAIVATLLALVPPGGHVVTTNDCYSEARAFIRDRL-SKMG
BdiCGS2  173 RYGNPTTEALEKKMSA----------LEKAESTVFVASGMYASVAMLSTLVPAGGHIVTTTDLYRKTRIYMETEL-PKRG
SbiCGS1  130 RYSNPTVKVLEDKISA----------LERAEATLVTSSGMNAITTTLLALVPPGGHVVTTTDCYSEARVFIRDKL-SRMG
SbiCGS2  182 RYGNPTTEALEKKMSA----------LERAESTVFVASGMYAAVAMLSALVPAGGHIVTTTDCYRKTRIYMETEL-PKRG
MacCGS1  178 RYGNPTTQALEEKMSA----------LERAESTLFVSSGMYASVAMLSSLVPAGGHIVITTDCYRKTRIFIENEL-PKMG
MacCGS2  174 RYGNPTTQALEEKMSA----------LERAESTLFVSSGMYASVAMFSALVPAGGHIVTTNDCYRKTRIFIESEL-PKMG
ZomCGS   248 RYGNPTSKVLEEKMSA----------LEKAESTLFVASGMHASVVMMQSLVPAGGHIITTTDCYRKTRMYIENQL-PMMN
AtrCGS   185 RYGNPTTKVLEEKISA----------LEEAESTVLMASGMCACTVMLMALVPAGGHIVTTTDCYRRTRIFIQTIL-PKMG
SmoCGS   131 RYGNPTTQATERKISA----------LEGAEETLVSASGMCAATTMLLALVPAGGHIVTTTDCYRRTRQFVQTVL-PKMG
SfaCGS1  236 RYGNPTTNAAEEKISA----------LEGAEATLLSASGMCVTTTMLLALVPAGGHIVTTTDCYRRTRQFIQTVL-PKMG
SfaCGS2  219 RYGNPTTNAAEEKISA----------LEGAESTLLSASGMCVTTTMLLALVPANGHIVTTTDCYRRTRQFIQTVL-PKMG
MpoCGS   210 RYGNPTTFAAESKISE----------LEGAETTLLSASGMCAATTMLLALVPAGGHIVTTTDCYRRTRQFIQTVL-PKMG
PpaCGS1  205 RYGNPTTNAVEEKISA----------LEGAETTLLSASGMCAATTMLLALVPAGGHIVTTTDCYRRTRQFIQTVL-PKMG
PpaCGS2  217 RYGNPTTNAVEEKISA----------LEGAEATLLSASGMCAATTMLLALVPAGGHIVTTTDCYRRTRQFIQTVL-PKMS
CreCGS   130 RYGNPTAQACENKIKA----------LEGAEDCLVSASGMNAVTSMLLSLVPAGGHIVTTSDCYWRTRQFMQNFL-PKMN
VcaCGS   137 RYGNPTTQACEAKIKA----------LEGAEDCLVSASGMNAVTSMLLSLVPSGGHIVTTSDCYWRTRQFMQNFL-PKMN
PglCGS   186 RYGNPTTQVAEEKISA----------LERAETTLLLASGMCATTTMMLALLPAGGHIITTTDCYRRTRQFIQTVL-PKMG
PsiCGS   186 RYGNPTTQVAEEKISA----------LERAETTLLLASGMCATTTMMLALLPAGGHIITTTDCYRRTRQFIQTVL-PKMG


AthCGS1  297 -ITVTVIDPAD-IAGLEAAVNEF------------------KVSLFFTESPTNPFLRCVDIELVSKICHKRGTLVCIDGT
AthCGS2  147 -ITVTFIDSDD-IAGLQTLVNNH------------------EVSLFFTESPTNPFLRCVDIKLVSKICHRRGTLVCIDAT
BraCGS1  264 -ITVTVIDPAD-IAGLEAAVNKY------------------QVSLFFTESPTNPFLRCVDIELVSEICHKRGTLVCIDGT
BraCGS2   45 -ITATLIDSTD-IAGLQAIVNNH------------------EVSMFFTESPTNPFLRCVDIKLVSEICHKRGTLVCIDGT
BraCGS3  232 --------------------------------------------------------------------------------
GmaCGS1  269 -ITTTVIDPAD-VGALESALEQH------------------NVSLFFTESPTNPFLRCVDIKLVSELCHKKGTLLCIDGT
GmaCGS2  270 -ITTTVIDPAD-VGALESALEQH------------------NVSLFFTESPTNPFLRCVDIKLVSELCHKKGTLLCIDGT
MdoCG1   257 -ITATIIDPAD-IGALETALEEH------------------KVSLFFTESPTNPFLRCVDIKLVSELCHKKGALVCIDGT
MdoCGS2  266 -ITATIIDPAD-VGALETALDEHKVS--------------EKVSLFFTESPTNPFLRCVDIKLVSELCHKKGALVCIDGT
MdoCGS3  257 -ITATIIDPAD-IGALETALEEH------------------KVSLFFTESPTNPFLRCVDIKLVSELCHKKGALVCIDGT
MtrCGS1  269 -ITTSVVDPAD-VGALQSALEQN------------------KVSLFFTESPTNPFLRCVDIKLVSELCHKNGALLVIDGT
MtrCGS2  211 -ITATIIDPAD-VDALEAALENN------------------KVSLFFTETPTNPFQRCVDIKLVSELCHRKGALVCIDGT
SlyCGS   274 -ITATVIDPAD-MGALELALNQK------------------KVDLFFTESPTNPFLRCVDIELVSKLCREKGALVCIDGT
VviCGS   231 -VEVTVIDPAD-TEALKSALDKN------------------NVTLFFTESPTNPFLRCVDIELVSELCHRKGALVCIDST
PvuCGS   274 -ITTTVIDPAD-VGALETALEQH------------------NVSLFFTESPTNPFLRCVDIKLVSELCHKKGALLCIDGT
TcaCGS   275 -ISATVIDPAD-VDGLEAALNKNKVSSVEIHPELKVHYDSIQVSLFFTESPTNPFLRCVDIEKVSKLCHSKGALVCIDGT
BolCGS1  270 -ISVTVIDPAD-IAGLEAAVNKY------------------QVSLFFTESPTNPFLRCVDIELVSEICHKRGTLVCIDGT
BolCGS2  244 -ITVTVIDPVD-TTRLEAAVNNH------------------EVSMFFTESPTNPFLRCVDIELVSEICHKRGTLVCIDGT
AcoCGS   281 -ITVTVIDPAD-IDGLQSALDQN------------------NVSLFFTESPTNPYLRCVDIELVSKICHSKGALVCIDGT
OsaCGS1  205 -ITSTFVDLDDDMEALESVLD-EDE-----------------VTMFYADSMTNPHLKVVDVARVAELCHRRGALVCIDST
OsaCGS2  219 -ITSTFVDLDDDMEALECVLD-ESE-----------------VTMFYADSMTNPHLKVVDVTRVAELCHRRGALVCIDST
OsaCGS3  150 -IRTTFVDLDD-MEALQSVLD-QGN-----------------VTMFYADSLTNPHLKCVDVRRVAELCHQRGALVCIDST
OsaCGS4  206 -ITSTFVDLDDDMEALESVLD-EGE-----------------VTMFYADSMTNPHLKVVDVTRVAELCHRRGALVCIDST
OsaCGS5  237 -ITMTVIRPAD-MDALQNALDNN------------------NVSLFFTETPTNPFLRCIDIDLVSKMCHSKGALLCIDST
ZmaCGS1   57 -ISMTVIRPAD-MDALQNALDNN------------------NVSLFFTETPTNPFLRCIDIEHVSNMCHSKGALLCIDST
ZmaCGS2  204 AIRSTFVDLDDDMESLKAVLDENDD-----------------VALFYADSPTNPLLKCVDIRHVAELCHRKGALVCIDST
ZmaCGS3  198 AIRSTFVDLDDGMKSLKAVLDENDD-----------------VALFYTDSPTNPMLKCVDIRHVAELCHRKGALVCIDST
ZmaCGS4  220 -ISMTVIRPAD-MDALQNALDNN------------------NVSLFFTETPTNPFLRCIDIEHVSNMCHSKGALLCIDST
SitCGS1  308 -VSMTVIRPAD-MDALQNALDNN------------------NVSLFFTETPTNPFLRCIDVELVANMCHSKGALLCIDST
SitCGS2  182 -IRSTFIDLDD-MESLKAVLE-QDD-----------------VTLFYADSPTNPLLKCVDIRLVAELCHRKGTLVCIDST
SitCGS3  182 -IRSTFIDLDD-MESLKAVLE-QDD-----------------VTLFYADSPTNPLLKCVDIRLVAELCHRKGTLVCIDST
BdiCGS1  201 -IRSTFVDLDD-IETLKAVLD-QGD-----------------VTLFYADSPTNPHLKCIDIKLVAELCHRKGALVCIDST
BdiCGS2  242 -ITMTVIKPAD-MNALQDALDKN------------------NVSLFFTETPTNPFLRCIDIELVSNMCHSKGALLCIDST
SbiCGS1  199 -IRSTFIDLDDGMESLSAVLDEDDDD----------------VTLFYADSPTNPTLKCVDIRHAAELCHRKGALVCIDST
SbiCGS2  251 -ISMTVIRPAD-MDALQNALDNN------------------NVSLFFTETPTNPFLRCIDIEHVSNMCHSKGALLCIDST
MacCGS1  247 -ISVTVIDPAD-IESLKISLDQN------------------NVSLFFSESPTNPFLRCVDIELVSQLCHNKGVLVCIDGT
MacCGS2  243 -ILATVIDPAD-TESLKSTLEQN------------------NVTLFFTESPTNPFLRCIDIELVSRLCHNNGALVCIDGT
ZomCGS   317 -ISATVIDPAD-IDGLKKALDEH------------------TVSLFFTESPTNPLFRCVDIELVSKLCHNKGTLVCVDST
AtrCGS   254 -ITATILDPAD-MDGLKSALEKH------------------KVSLYFTESPTNPFLRCVDIELVSKLCHSKGAVVCIDGT
SmoCGS   200 -ITTTVIDPAD-MASLKRALDDN------------------PVSLFFSESPTNPYLRCIDIELVSTLCRRKGAIVCIDGT
SfaCGS1  305 -ITTTVIDPAD-ISALQHALETK------------------NVSLFFSESPTNPYLRCIDVELVSKLCHTHGVLVCIDGT
SfaCGS2  288 -ITTTVIDPAD-TATLQRALETK------------------NVSLFFSESPTNPYLRCIDVELVSKLCHAHGALVCIDGT
MpoCGS   279 -ITTTVIDPSD-IASLERALDQH------------------TVSLFFSESPTNPYLRCIDIELVSKLCHSKGTLVCIDGT
PpaCGS1  274 -ITTTVIDPAD-ISALQHALEQN------------------KVSIFFSESPTNPYLRCIDIELVSKLCHEHGALVCIDGT
PpaCGS2  286 -ITTTVVDPAD-ISSLQLALEQN------------------NVSIFFSESPTNPYLRCIDVELVSKLCHEHGALVCIDGT
CreCGS   199 -IGVSVIKPND-LEALQEALDKH------------------SVTLFFSESPTNPYLRCVDILAISRLCHAKGAFVCVDST
VcaCGS   206 -VGVSVIKPND-LAALQQALDQH------------------NVTLFFSESPTNPYLRCVDIPAIARLCHAKGAAVCIDST
PglCGS   255 -IKATVIDPAD-IDSLELALEEN------------------NVSLFFSESPTNPYLRCIDIELVSKICHRKGVLVCIDCT
PsiCGS   255 -IKATVIDPAD-IDSLELALEEN------------------NVSLFFSESPTNPYLRCIDIELVSKICHRKGVLVCIDCT

AthCGS1  357 FATPLNQKALALGADLVVHSATKYIGGHNDVLAGCICGSLKLVSEIRNLHHVLGGTLNPNAAYLIIRGMKTLHLRVQQQN
AthCGS2  207 IATPINQKTLALGADLVHHSATKYIGGHNDFLAGSISGSMELVSKIRNLHKLLGGTLNPNAAYLLIRGMKTMHLRVRQQN
BraCGS1  324 FATPLNQKALAFGADLVVHSLTKYIGGHNDVLGGCICGPLKVVSEIRNLHHVLGGTLNPNAAYLMIRGMKTMHLRVQQQN
BraCGS2  105 IATPLNQKALALGADLVIQSATKYIGGHNDVLAGCISGSMKLVSEIRNMHNLLGGTLSPNAAYLLIRGIKTMHLRVKQQN
BraCGS3  232 --------------------------------------------------------------------------------
GmaCGS1  329 FATPLNQKALALGADLILHSLTKYMGGHHDALGGCISGSTKVVSQIRTLHHVLGGTLNPNAAYLFIRGMKTLHLRVQQQN
GmaCGS2  330 FATPLNQKALALGADLILHSLTKYMGGHHDVLGGCISGSIKVVSQIRTLHHVLGGTLNPNAAYLFIRGMKTLHLRVQQQN
MdoCG1   317 FATPLNQKALALGADLVVHSATKYIGGHNDVLAGCISGSMKLVSEIRILHHILGGALNPNAAYLIIRGMKTLHLRVQQQN
MdoCGS2  330 FATPLNQKALALGADLVVHSATKFIGGHNDVLAGCISGSMKLISEIRTLHHVLGGALNPNAAYLIIRGMKTLHLRVQQQN
MdoCGS3  317 FATPLNQKALALGADLVVHSATKYIGGHNDVLAGCISGSMKLVSEIRILHHILGGALNP---------------------
MtrCGS1  329 FATPLNQKALALGADLVMHSCTKYIGGHHDVLGGCISGSLKLISEIRILHHILGGALNPNAAYLFIRGMKTLHLRVQQQN
MtrCGS2  271 FSTPINQKALSLGADLVVNSATKYIAGHHDVLAGCISGSEKLISQVRSFHFILGGTLSPNSAYLVIRGMKTLHLRVQHQN
SlyCGS   334 FATPLNQKALALGADLVVHSATKFLGGHNDVLAGCISGPEKLVSVIRNLHHILGGALNPNAAYLIIRGMKTLHLRVQQQN
VviCGS   291 FATPLNQKTLSLGADLVLHSATKYIAGHNDVIAGCISGSEKLVSTIRNLHHVLGGVLNPNAAYLIIRGMKTLHLRVQQQN
PvuCGS   334 FATPLNQKALALGADLIMHSLTKYMSGHHDVLGGCISGSTKVVSQIRTFHHILGGTLNPNAAYLLIRGMKTLHLRVQQQN
TcaCGS   353 FATPLNQKVLALGADLVLHSATKFIGGHNDVLAGCVSGSEKLITEIRTLHHILGGTLNPNAAYLIIRGMKTLHLRVQQQN
BolCGS1  330 FATPLNQKALAFGADLVVHSLTKYIGGHNDVLGGCICGPLKVVSEIRNLHHVMGGTLNPNAAYLIIRGMKTMHLRVQQQN
BolCGS2  304 LATPLNQKALALGADLVVHSATKYIGGHNDVLGGCVCGSMELVSAIRNLHHVLGGTLNPNAAYLLIRGIKTMHLRLKQQN
AcoCGS   341 FATPLNQKALSLGADIVLNSATKYIGGHNDVIGGCISGSEEVVSKIRKLHHVLGGVLNPNAAYLFIRGIKTLHVRIKQQN
OsaCGS1  266 LASPINQKPLALGADVVLHSATKYIAGHHDVIAGCVSGSEALISRIRAWHHDLGGAISPNAAYMIIRGLKTLALRVEAQN
OsaCGS2  280 LASPINQKPLALGADVVLHSATKYIAGHHDVIAGCVSGSEALISRIRAWHHDLGGAISPNAAYMIIRGLKTIALRVEAQN
OsaCGS3  210 LASPINQKPLTLGADVVLHS-------HHQVIG--AGWETPWVTDFTTGMGVGFLNWHPNAAYMIIRGLKTMALRVEAQN
OsaCGS4  267 LASPINQKPLALGADVVLHSATKYIAGHHDVIAGCVSGSEALVSRIRTWHHDLGGAISPNAAYMIIRGLKTLALRVQAQN
OsaCGS5  297 FASPINQKALTLGADLVIHSATKYIAGHNDVIGGCISGRDELVSKVRIYHHVVGGVLNPNAAYLILRGMKTLHLRVQCQN
ZmaCGS1  117 FASPINQKALTLGADLVIHSATKYIAGHNDVIGGCVSGRDELVSKVRIYHHVVGGVLNPNAAYLILRGMKTLHLRVQCQN
ZmaCGS2  267 MASPINQKPLTLGADLVLHSATKYMAGHHDVIAGCISGSEALISRIRAWHHQLGGAISPNAAYTVIRGLKTMALRVEAHN
ZmaCGS3  261 MASPINQKPLTLGADLVLHSAIKYMAGHHDAIAGCVSGSEALITRIRAWHHHLGGVISPSAAYMVIRGLKTMALRVEAHN
ZmaCGS4  280 FASPINQKALTLGADLVIHSATKYIAGHNDVIGGCVSGRDELVSKVRIYHHVVGGVLNPNAAYLILRGMKTLHLRVQCQN
SitCGS1  368 FASPINQKALTLGADLVIHSATKYIAGHNDVIGGCVSGRDELVSKVRIYHHVVGGVLNPNAAYLILRGMKTLHLRVQCQN
SitCGS2  242 LASPINQKPLTLGADVVLHSATKYMAGHHDVIAGCISGSKTLISKIRAWHHDLGGAISPNAAYMIIRGLKTMALRVEAHN
SitCGS3  242 LASPINQKPLTLGADVVLHSATKYMAGHHDVIAGCISGSKTLISKIRAWHHDLGGAISPNAAYMIIRGLKTMALRVEAHN
BdiCGS1  261 LSSPINQKPLTLGADIVVHSATKYIAGHHDVIAGCISGSDALLSRIRAWHHDLGGAISPDAAYMIIRGLKTMALRVETQN
BdiCGS2  302 FASPINQKALPLGADIVVHSATKYIAGHNDVIGGCISGRDELVSKVRIYHHVVGGVLNPNASYLILRGMKTLHLRVQCQN
SbiCGS1  262 MASPINQKPLTLGADLVLHSATKYMAGHHDAIAGCVSGSAALISRIRAWHHDIGGAISPNAAYMVIRGLKTMALRVEAHN
SbiCGS2  311 FASPINQKALTLGADLVIHSATKYIAGHNDVIGGCVSGRDELVSKVRIYHHVVGGVLNPNAAYLILRGMKTLHLRVQCQN
MacCGS1  307 FASPVNQKALTLGADLVLHSATKFIGGHNDVLGGCISGSEELISKISLHHHVIGGVLNPNAAYMILRGMKTLHLRVQNQN
MacCGS2  303 FASPVNQKALALGADLILHSATKFIAGHNDVIGGCISGSEELISKIQLYHNVVGGVLNPNDAYMILRGMKTLHLRVQNQN
ZomCGS   377 FASPINQKALTFGADLVLHSATKFLNGHNDVLGGCISGSMEVINKIRKLHHVIGGVINPNASYMILRGMKTLKLRVQQQN
AtrCGS   314 FATPVNQKALPLGADLVVHSATKYIAGHNDVIAGCISGSKKLVETVRALHHVLGGVLNPNAAYMIIRGMKTLHLRVQQHN
SmoCGS   260 FATPVNQKALALGADLVLNSATKYIAGHNDVLAGSISGSKPLVDTVRKLHNVLGGVVDAHAAYLIGRGLKTLDLRVRQQN
SfaCGS1  365 FATPVNQQALALGADLVLNSATKYLAGHNDVLAGSLSGSKKCLTPVRALHNILGGVLDPNAAYLILRGLKTLTIRVRQQN
SfaCGS2  348 FATPVNQQALALGADLVLQSATKYLAGHNDVLAGSLSGSKKCLTPVRALHNVLGGVLDPNAAYLILRGLKTLHIRVRQQN
MpoCGS   339 FATPVNQKALALGADIVLHSATKYLAGHNDVLAGSVSGSKECIAPIRALHHILGGVLDPNSAYLILRGMKTLELRVRQQN
PpaCGS1  334 FATPVNQQALALGADLVLHSATKYLAGHNDVLAGSLSGSKQCLSAVRALHNILGGVVDPNAAYLILRGLKTLDLRVKQQN
PpaCGS2  346 FATPVNQQVLALGADLVLHSATKYLAGHNDVLAGSLSGTKQCLSAVRALHNILGGVVDPNAAYLILRGLKTLDLRVKQQN
CreCGS   259 FATPINTRALELGADLVLHSATKYLAGHNDVLAGALCGKKELVEKVREFHHIMGGVVDPHAAYLLLRGLKTLELRVTRHN
VcaCGS   266 FATPINQQALALGADLVLHSATKYLAGHNDVLAGALCGKPELVGKVREFHHIMGGVVDPHAAYLLLRGLKTLDLRVERHN
PglCGS   315 FATPVNQKALALGADIVLHSVTKYIAGHNDVLAGSLSGSNKIIGAVRALHNVLGGVLSPNAAYLILRGIKTLHLRVQQQN
PsiCGS   315 FATPVNQKALALGADIVLHSVTKYIAGHNDVLAGSLSGSNKIIGAVRALHNVLGGVLSPNAAYLILRGIKTLHLRVQQQN


AthCGS1  437 STAFRMAEILEAHPKVSHVYYPGLPSHPEHELAKRQMTGFGGVVSFEI------------DGDIETTIKFVDSLKIPYIA
AthCGS2  287 STGMKMAQVLEAHPKVSRVYYLGLPSHPEHLIAKRQMTGIGGLISFEI------------DGDLKTTIKFIDALKIPYLA
BraCGS1  404 STASRMAEILEAHPKVSHVYYPGLASHPEHHIAKRQMTGFGGVVSFEI------------DGDIERTIKFVDSLKIPYIA
BraCGS2  185 STALRMAHVLEAHPKVSRVYYPGLPNHPEHHIAKRQMTGFGGLVTFEI------------DGDLETTIKFIDSLKIPYIA
BraCGS3  232 --------------------------------------------------------------------------------
GmaCGS1  409 STGMRMAELLEAHPKVKRVYYPGLPSHPEHELAKRQMTGFGGVVSFEI------------DGDLHTTIKFIDSLKIPYIA
GmaCGS2  410 STGMRMAKLLEAHPKVKRVYYPGLPSHPEHELAKRQMTGFGGVVSFEI------------DGDLHTTIKFIDSLKIPYIA
MdoCG1   397 STALRMAKILEAHPKVAHVYYPGLPSHPEHQLAKRQMTGFGGVVSFEI------------DGDLTRTINFVDALKIPYIA
MdoCGS2  410 STALRMAKILEAHPKVAHVYYPGLPSHPEHQLAKRQMTGFGGVVSFEVNILGAIPFWYVIDGDLMRTIKFVDALKIPYIA
MdoCGS3      --------------------------------------------------------------------------------
MtrCGS1  409 STGMRMAKLLEAHPKVKRVYYPGLPSHPEHELAMRQMTGFGGVVSFEI------------DGDITTTIKFIDSLKIPYIA
MtrCGS2  351 STAMKMAQLLETHPKVTRVYYPGLPSHPEYALAKRQMTGFGGVVCFDI------------DGDIMRTIKFVDSLKIPYIA
SlyCGS   414 STALRMAEILEAHPKVKHVYYPGLPSHPEYHLAKKQMTGFGGVVSFEV------------DGDLLTTAKFVDALRIPYIA
VviCGS   371 STALRMAKILEAHPKVKCVYYPGLPSHPEHHIAKRQMTGFGGVVSFEV------------DGDLTTTIKFVDALKIPYIA
PvuCGS   414 STGMGMAKILEAHPKVKRVYYPGLPSHPEHELAKRQMTGFGGVVSFEI------------DGDIHTTIKFVDSLKIPYIA
TcaCGS   433 STALKMAKVLEAHPRVKRVYYPGLPSHPEHEIAKLQMTGFGGVVSFEV------------DGDLMTTIKFVDALKIPYIA
BolCGS1  410 STASRMAETLEAHPKVSHVYYPGLANHPEHHIAKRQMTGFGGVVSFEI------------DGDIERTIKFVDSLKIPYIA
BolCGS2  384 STALRMAQVLEAHPKVSRVYYPGLISHPEHHIAKRQMNGFGGVVSFEI------------AGDLETTIKFVDSLNIPYIA
AcoCGS   421 STALEMAKILEAHPKIVRVHYPGLPSHPDHDIAMQQMTGFGGVVSFEV------------DGDLHTTKKFIDALKIPYIA
OsaCGS1  346 RTALRMARLLEKHPKIERVYYPGLESSPWHGVATRQMAGAGGVVSFEV------------ASDMRGVMRFVDALELPLIA
OsaCGS2  360 RTALRMARLLEKHPKIERVYYPGLESSLWHGVATRQMAGAGGVVSFDV------------ASDLRGVMRFVDALELPLIA
OsaCGS3  281 RTALRVARLLERHPKVERVNYPWLESSPWHGVARKQMTGAGGVISFEV------------ASDMRGAMRFVDALELPFIA
OsaCGS4  347 RTALRMAQLLEKHPKIERVYYPGLESSPWHGVATRQMAGAGGVVGLEV------------ASDMRGAMRFVDALELPLIA
OsaCGS5  377 NTAMRMAQFLEEHPKIARVYYPGLPSHPEHHIAKSQMTGFGGVISFEV------------AGDFDATRRFIDSVKIPYHA
ZmaCGS1  197 DTALRMAQFLEEHPKIARVYYPGLPSHPEHHIAKSQMTGFGGVVSFEV------------AGDFDATRKFIDSVKIPYHA
ZmaCGS2  347 STALRMARMLERHPKIERVHYPGLESSPWHEVARTQMSGYGGVVRLEL------------KADLRGTMSFVDALEIPFIA
ZmaCGS3  341 STALRMARMLERHPKIERVHYPGLESSPWHELARTRMSGYGGVVSFEL------------KADLRGTMRFVDALEIPFIA
ZmaCGS4  360 NTALRMAQFLEEHLKIARVYYPGLPSHPEHHIAKSQMTGFGGVVSFEV------------AGDFDATRKFIDSVKIPYHA
SitCGS1  448 NTAMRMAQFLEEHPKIARVYYPGLPSHPEHHIAKTQMTGFGGVVSFEV------------AGDFDGTRRFIDSVKIPYHA
SitCGS2  322 RTALDMARLLELHPKIERVHYPGLESNPWHQVAKSQMTGYGGVVSFEV------------KSDLCGTMRFVDALEIPLIA
SitCGS3  322 RTALDMARLLELHPKIERVHYPGLESNPWHQVAKSQMTGYGGVVSFEV------------KSDLCGTMRFVDALEIPLIA
BdiCGS1  341 RTALRMARLLENHPKIERVYYPGLKSSPWHHVAKSQMTGFGGVISFEV------------ASDLRGVMRFVDALEIPFIA
BdiCGS2  382 NTALRMAQFLEEHPKIARVYYPGLPSHPEHHIAKSQMTGFGGVVSFEV------------NGDFDSTRKFIDSVKIPYHA
SbiCGS1  342 STALRMARILERHPKIERVHYPGLESSPWHEVATSQMSGYGGVVSFEL------------KADLHGTMSFVDALEIPFIA
SbiCGS2  391 NTALRMAQFLEEHPKIARVYYPGLPSHPEHHIAKSQMTGFGGVVSFEV------------AGDFDGTRRFIDSVKIPYHA
MacCGS1  387 STALRMAQFLEEHPKIIRVYYPGLPSHPEHHIAKRQMTGFGGVVSFEV------------AGDLNTTKKFVDSLKIPYIA
MacCGS2  383 STALRMAQLLEEHPKIIHVYYPGLPSHPEHHIAKCQMTGFGGVVSFEI------------AGDLSTTKKFIDSLKIPYIA
ZomCGS   457 TTAMRMAQFLEDHPKISRVYYPGLPSHPEHDIAKKQMSGFGGVVSFEV------------EGDLKITSKFVDSLNIPYNA
AtrCGS   394 LTAMRMARLLEAHPKITRVYYPGLPSHPEHHIASRQMTGFGGVVSFEV------------AGDLNTTAKFVDALKIPYIA
SmoCGS   340 SNALRLARALESHPKIARVHYPGLESHPEFHIASKQMRGFGGVVSFEI------------DGDLALTSKFIDGLRIPYIA
SfaCGS1  445 ATALKLAQTLEAHPKVARVHYPGLESHPEHNIAKLQMSGFGGVVSFEI------------VGDLETTSRFIDGLRIPYIA
SfaCGS2  428 ATAIKLARTLEAHPKVVRVHYPGLESHPEHSIAKRQMSGFGGVVSFEI------------VGDLETTSRFIDGLRIPYIA
MpoCGS   419 ISALKLAQALEAHPKVLRVHYPGLESHPEHHIAKRQMSGFGGVISFEI------------AGGLDEASKFIDGLKIPYIA
PpaCGS1  414 KTAYLLAQNLEAHPKVARVHYPGLESHPEHQIAKRQMTGFGGVISFEI------------DGDLETTSKFIDGLRIPYIA
PpaCGS2  426 RTALLLAQKLEAHPKVARVHYPGLESHPEHQIAKRQMSGFGGVISFEI------------NGDLETTSNFIDGLRIPYIA
CreCGS   339 ASAMEIARRLESHPKVVRVWYPGLESHPDHAIAKRQMSGFGGVVSFET------------RGGLNECISFIDNVKLPYIA
VcaCGS   346 RSAMEIARRLEQHPKIDRVWYPGLESHPDHAIAKRQMSGFGGVVSFEV------------RGGLQECISFIDNVKLPYIA
PglCGS   395 STALRIARTLEAHPKVSRVHYPGLESHPEHHIAKRQMSGFGGVVSFEI------------VGDLQATSRFIDSLKIPYIA
PsiCGS   395 STALRIARTLEAHPKVSRVHYPGLESHPEHHIAKRQMSGFGGVVSFEI------------VGDLQATSRFIDSLKIPYIA

AthCGS1  505 PSFGGCESIVDQPAIMSYWDLPQE-ERLKYGIKDNLVRFSFGVEDFEDVKADILQALEAI----------------
AthCGS2  355 ASFGGCESLVDQLATGI-WDIPRE-ERLKDGFQDNLVRFSFGIEDFEDIKADVLQALETI----------------
BraCGS1  472 PSFGGCESIVDQPAIMSYWDLTQE-ERLKYGIKDNLVRFSFGIEDFEDVKADVLQALEAI----------------
BraCGS2  253 TSFGGCESFVDQPATRN-WDVPQE-ERLKYGHKDNLVRFSFGVEDFEDLKADILQALETTPTKTSSVSHQNGAVFD
BraCGS3  232 --------IVDQPAIMSYWDLAPE-ERLKYGIKDNLVRFSFGVEDFEDVKADVLEALEAI----------------
GmaCGS1  477 ASFGGCESIVDQPAILSYWDLPQS-ERAKYKIYDNLVRFSFGVEDFEDLKADVLQALEAI----------------
GmaCGS2  478 ASFGGCESIVDQPAILSYWDLPQS-ERAKYKIYDNLVRFSFGVEDFEDLKADVLQALEAI----------------
MdoCG1   465 PSFGGCESIVDQPAIMSYWDLSQS-DRIKYGIKDNLVRFSFGVEDFEDLKADILQALETI----------------
MdoCGS2  490 PSFGGCESIVDQPAIMSYWDLSQX-DRIKYGIKDNLVRFSFGVEDFEDLKADILQALETI----------------
MdoCGS3      ----------------------------------------------------------------------------
MtrCGS1  477 ASFGGCESIVDQPAILSYWDLPAS-ERAKWKIYDNLVRFSFGIEDFEDLKADVLQALEAI----------------
MtrCGS2  419 PSFGGVESVVNQPAIMSYWDLPRK-EREKYRIYDNTVRFCFGVEGYEDLKDDVLQALDAI----------------
SlyCGS   482 PSFGGCESIVDQPAIMSYWDLSQS-DRAKYGILDNLVRFSFGVEDFEDVKADVLQALDSI----------------
VviCGS   439 PSFGGCESIVDQPAIMSYWDLNQS-ERAKYGIQDNLVRFSFGVEDFEDLKADILQALESI----------------
PvuCGS   482 ASFGGCESIVDQPAILSYWDLPQS-ERAKYKIHDNLVRFSFGVEDFEDLKADVLQALEAI----------------
TcaCGS   501 PSFGGCESIVDQPAIMSYWDLTQA-ERRKYGIEDNLVRFSFGVEDFEDLKADILQALETI----------------
BolCGS1  478 PSFGGCESIVDQPAIMSYWDLAPE-ERLKYGIKDNLVRFSFGVEDFEDVKADVLQALEAI----------------
BolCGS2  452 PSFGGCESIVDQPAIMSYWDLPQE-ERLKYGIKDNLVRFSFGIEDFDDVKADVLQALGII----------------
AcoCGS   489 PSFGGCESIVDQPAIMSYWDLTQD-GRAMFGIKDNLVRFSFGLEDFEDLKADILQALATL----------------
OsaCGS1  414 TSLGGCESLVQQPAVMSYWGKSEE-EKAKNGIKDNFVRFSFGIEKFEDLRDDILQALEKI----------------
OsaCGS2  428 TSLGGCESLVQQPAVMSYWGKSEE-EKAKNGIKDNFVRFSFGIEKFEDLRDDILQALEKI----------------
OsaCGS3  349 TSLGGCESLVQQPAIMSYWGKSEA-EKTENGIKDNLVRFSFGIEKFEDLKDDILQALEKI----------------
OsaCGS4  415 TSLGGCESLVQQPAIMSYW---------------------------------------------------------
OsaCGS5  445 PSFGGCESIIDQPAIMSYWDSKE--QREIYGIKDNLIRFSIGVEDFEDLKNDVVQALDKI----------------
ZmaCGS1  265 PSFGGCESIIDQPAIMSYWDSKE--QRDIYGIKDNLIRFSIGVEDFEDLKNDLVQALEKI----------------
ZmaCGS2  415 TSLGGCESLVQQPAVMSFWGRSQE-DKTKNGIKDNLVRFSFGIEKFEDLQDDILQALNRI----------------
ZmaCGS3  409 TSLGGCESLVQQPAVMSFWGHSEE-DKAKNGIKDNLVRFSFGIEKFEDLRDDILQALNKI----------------
ZmaCGS4  428 PSFGGCESIIDQPAIMSYWDSKE--QRDIYGIKDNLIRFSIGVEDFEDLKNDLVQALEKI----------------
SitCGS1  516 PSFGGCESIIDQPAIMSYWDSKE--QRDIYGIKDNLIRFSIGVEDFEDLKNDLVQALEKV----------------
SitCGS2  390 TSLGGCESLVQQPAVMSFWGKSDD-EKAKNGIKDNLVRFSFGIEKFEDLRDDILQALEKI----------------
SitCGS3  390 TSLGGCESLVQQPAVMSFWGKSDD-EKAKNGIKDNLVRFSFGIEKFEDLRDDILQALEKI----------------
BdiCGS1  409 TSLGGCESLVQQPAVMSFWGQSDE-EKSKNGITDNLVRFSFGIEKFEDLRDDILQALEKV----------------
BdiCGS2  450 PSFGGCESIIDQPAIMSYWDSKE--QRDIYGIKDNLIRFSVGVEDFEDLKNDIVQALDKI----------------
SbiCGS1  410 TSLGGCESLVQQPAVMSFWGQSQE-EKAKNGIKDNMVRFSFGIEKFQDLRDDILQALNRI----------------
SbiCGS2  459 PSFGGCESIIDQPAIMSYWDSKE--QRDIYGIKDNLIRFSIGVEDFEDLKNDLVQALEKI----------------
MacCGS1  455 PSFGGCESIVDQPAIMSYWDLSGSERVAKYGIKDNLVRFSFGVEGFEDLKADILQALEKI----------------
MacCGS2  451 PSFGGCESIIDQPAIMSYWDLSRPERTAKYGIKDNLVRFSFGVEGFEDLNADNLQSLEKI----------------
ZomCGS   525 PSFGGCESIIDQPAIMSYWDLPAP-ERIKYGIRDNLIRFSLGIEDFEDLKEDISQALEKI----------------
AtrCGS   462 PSFGGCESIIDQPAIMSYWDLSPV-ERAQYGIKDNLVRFSLGIEDFEDLRMDVLQALDSI----------------
SmoCGS   408 PSLGGCESLVEQPTIISYWDQSPA-ERAKMGIKDNLVRFSCGIEDYEDIYADVMQSLASL----------------
SfaCGS1  513 PSLGGCESLVEQPTIISYWDQTPE-ERAKLGIKDNLVRFSCGIEDSEDIFHDIMQSLDAM----------------
SfaCGS2  496 PSLGGCESLVEQPTIISYWDQTPA-ERAKLGIKDNLVRFSCGIEDSEDIFHDILQSLDAL----------------
MpoCGS   487 PSLGGCESLVEQPTIISYWDQTAA-QRAKLGIKDNLVRFSCGIEDYEDIHNDMMQSLAAL----------------
PpaCGS1  482 PSLGGVESLVEQPTIISYWDQSPA-ERARIGIKDNLVRFSCGVEDYEDILNDVMQSLDAL----------------
PpaCGS2  494 PSLGGVESLVEQPTIISYWDQSPA-ERARLGIKDNLVRFSCGIEDYEDILNDVMQSLNAL----------------
CreCGS   407 PSLGGVESLIEMPAVQSYWGFGPE-RRAQIGIKENLIRFSIGVEDVEDIWADLVQALEYVP---------------
VcaCGS   414 PSLGGVESLIEMPAVQSYWGFGPE-RRAQIGIKENLVRFSIGIEDVDDIWADLVQALEYVP---------------
PglCGS   463 PSLGGCESLVEQPTIISYWDQSSE-ERARLGIKDNLVRFSCGVEAFDDIESDVLQALEAV----------------
PsiCGS   463 PSLGGCESLVEQPTIISYWDQSSE-ERARLGIKDNLVRFSCGVEAFDDIESDVLQALEAV----------------


Note: N-terminal extended region (amino acids 1-173 in AthCGS1) , the chloroplast targeting region (amino acids 27-136 in AthCGS1), the conserved region  (amino acids 58-98 in AthCGS1) and MTO1(amino acids 77-87 in AthCGS1) in CGS proteins was marked by the blue, green, orange and red lines.
Three key sites (R77, S81, G84 in AthCGS1) in MTO1 region were marked by the black boxes.
