# Supplementary material for: Molecular evolution and expression divergence of three key Met biosynthetic genes in plants: CGS, HMT and MMT
Source: PeerJ. 2018 Dec 4;6:e6023. doi: 10.7717/peerj.6023 (PMC6284425; doi:10.7717/peerj.6023)
Supplement: Supplemental Information 2 [file peerj-06-6023-s002.pdf]

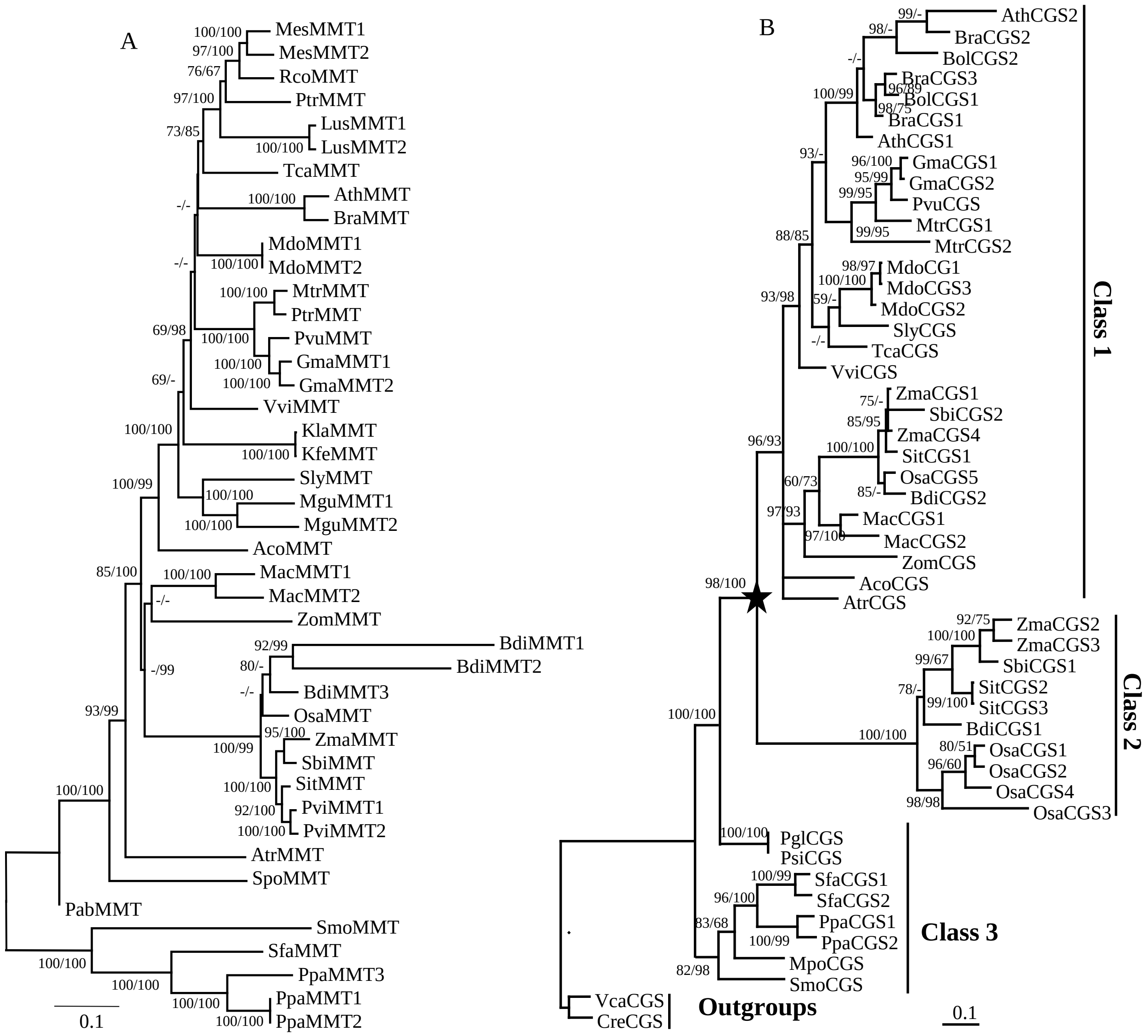

Figure S1 Phylogenetic tree of the *MMT* (A) and *CGS* (B) gene family in plants. The trees were constructed with maximum likelihood (ML) and neighbor-joining (NJ) methods based on the amino acid (aa). Support values (>50% of ML and NJ) for the two trees are shown on respective branches. The black star indicates the divergence of Class 1 and Class 2. Gene names and identifiers are shown in Table S1A,B.

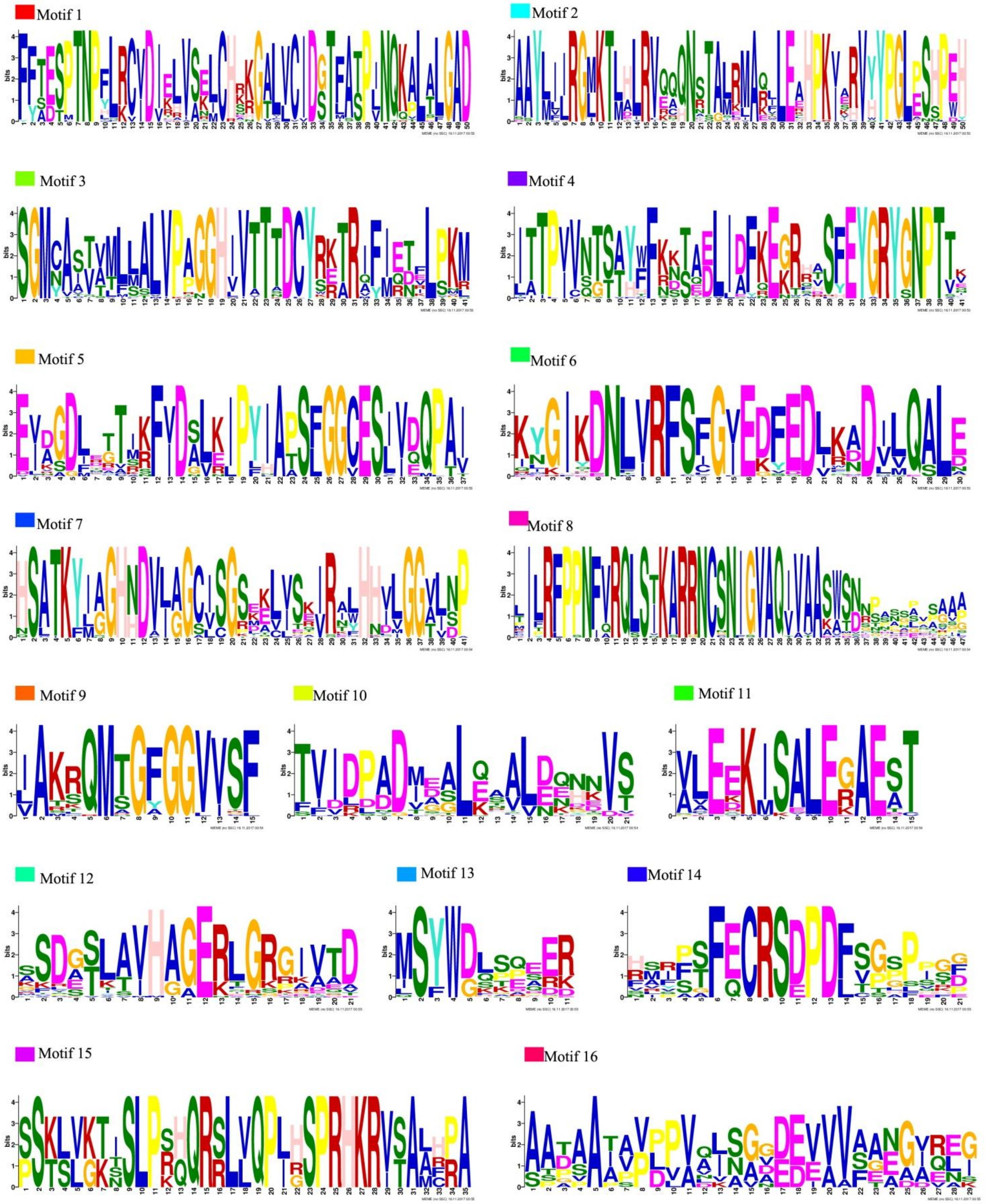

Figure S2A. Sequences of the conserved motifs detected by the MEME analysis on the CGS homologs across plants. The height of the each letter denotes the probability of the letter at that position, and total height of the stack represents the information content of that position.

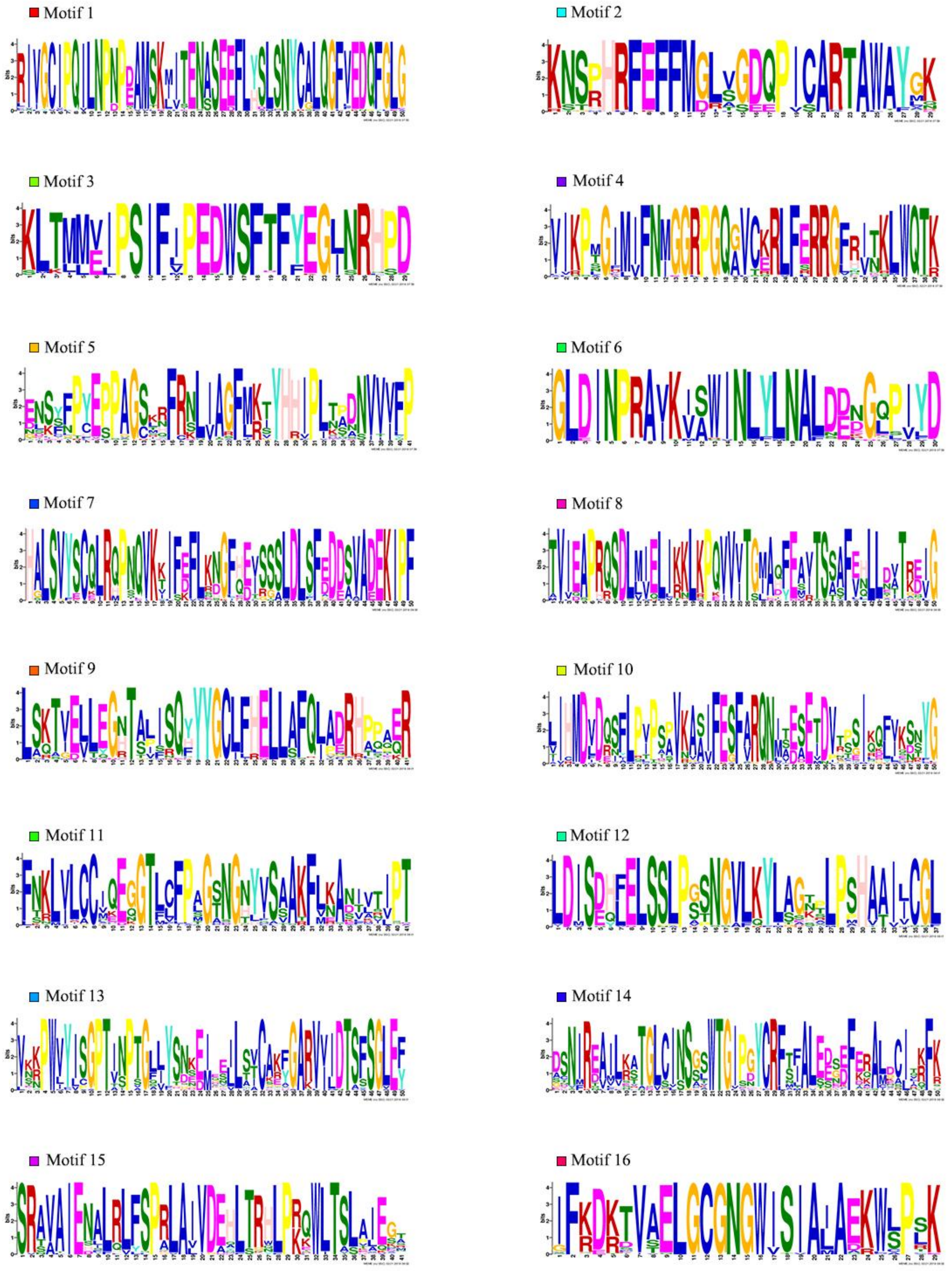

Figure S2B. Sequences of the conserved motifs detected by the MEME analysis on the MMT homologs across plants. The height of the each letter denotes the probability of the letter at that position, and total height of the stack represents the information content of that position.

Figure S3 Zhao et al. 2018

AthCGS1

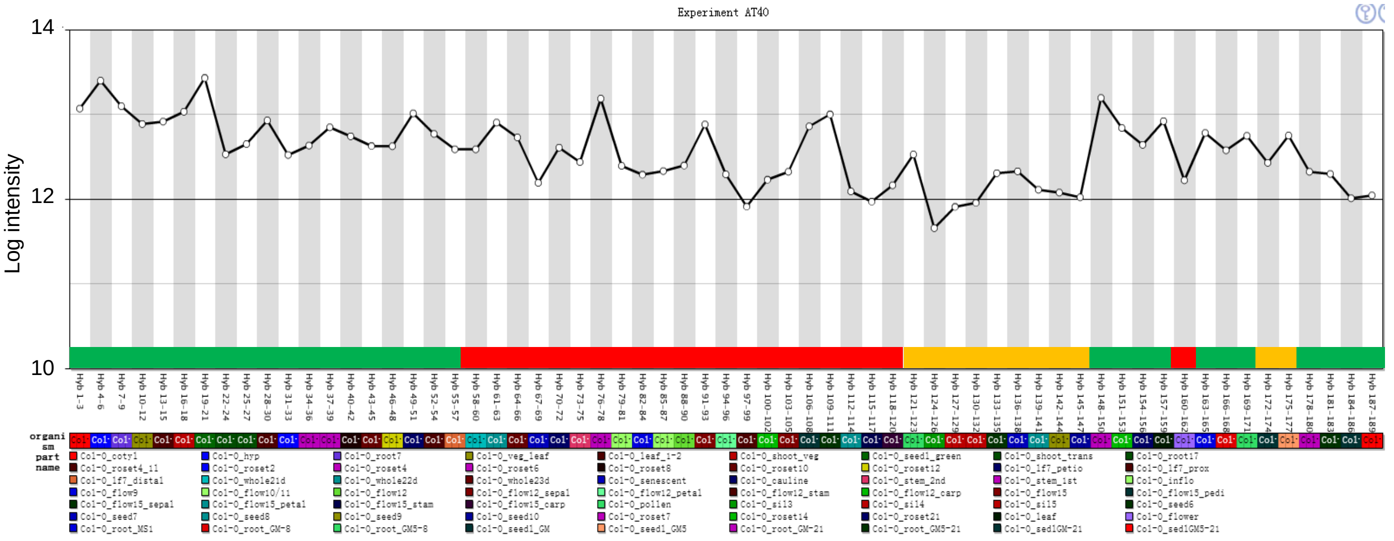

AthCGS2

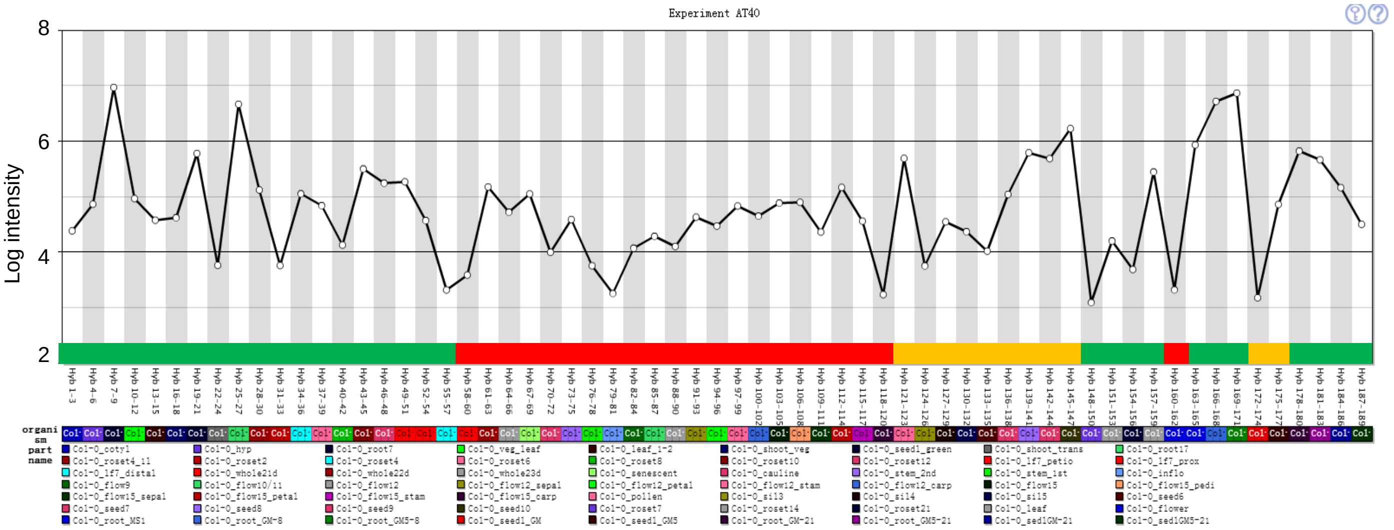

Figure S3A. Tissue-specific expression data of *AthCGS*s from PLEXdb.
The green, red, orange boxes represent vegetative tissues such as roots, stems and leaves, flowers, and seeds, respectively.

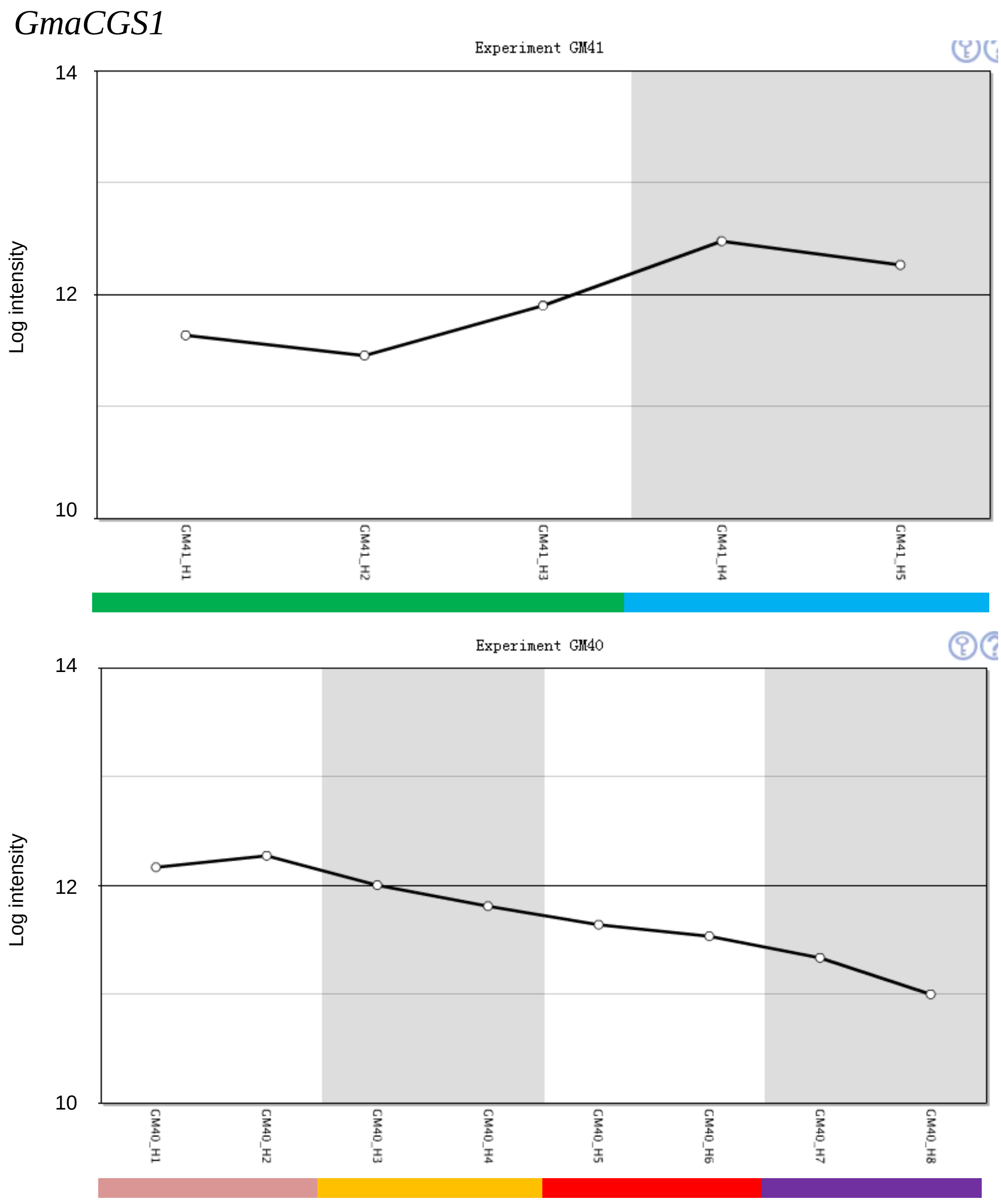

Figure S3B. Tissue-specific expression data of *GmaCGS1* from PLEXdb.
The green and blue boxes represent root apical meristem and leaf non-meristem. The pink, orange, red and purple boxes represent bean 2mm, bean 5mm, pod elongation and fully grown, respectively .

OsaCGS1

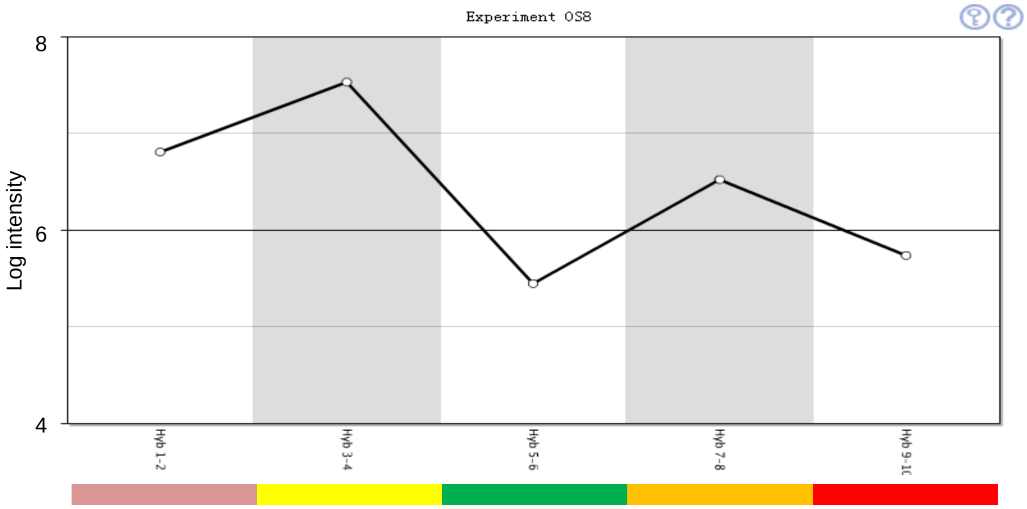

OsaCGS3

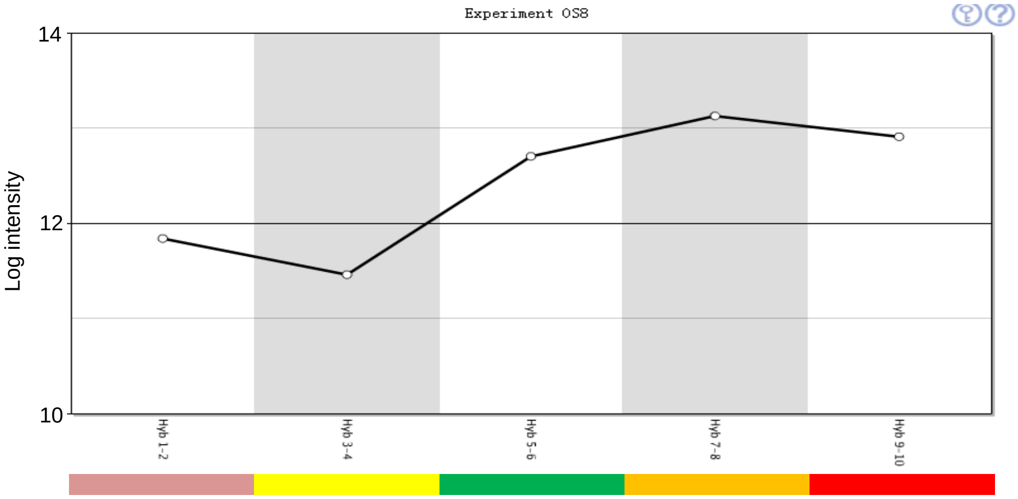

OsaCGS5

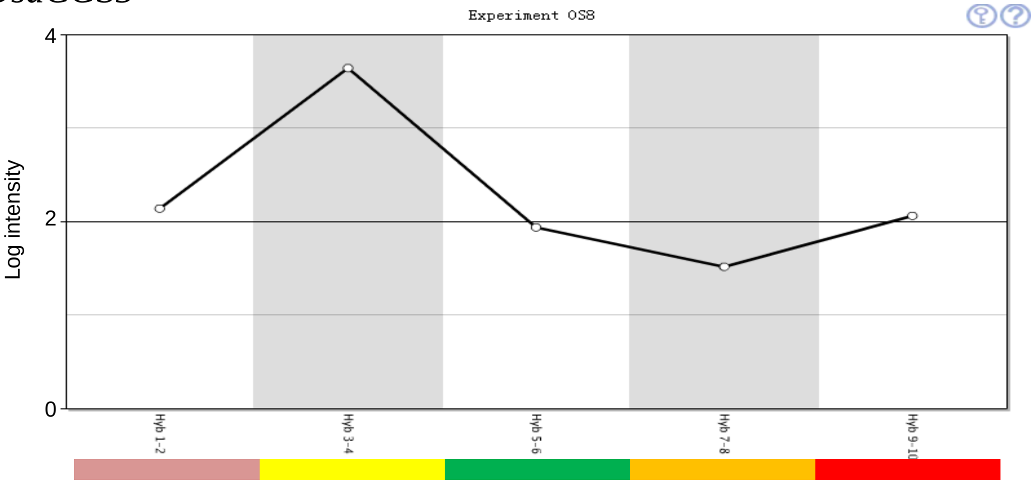

Figure S3C. Tissue-specific expression data of *OsaCGS*s from PLEXdb.
The pink, yellow, green, orange and red boxes represent emb6D, endo6D, leaf, root and seedling, respectively.

AthMMT

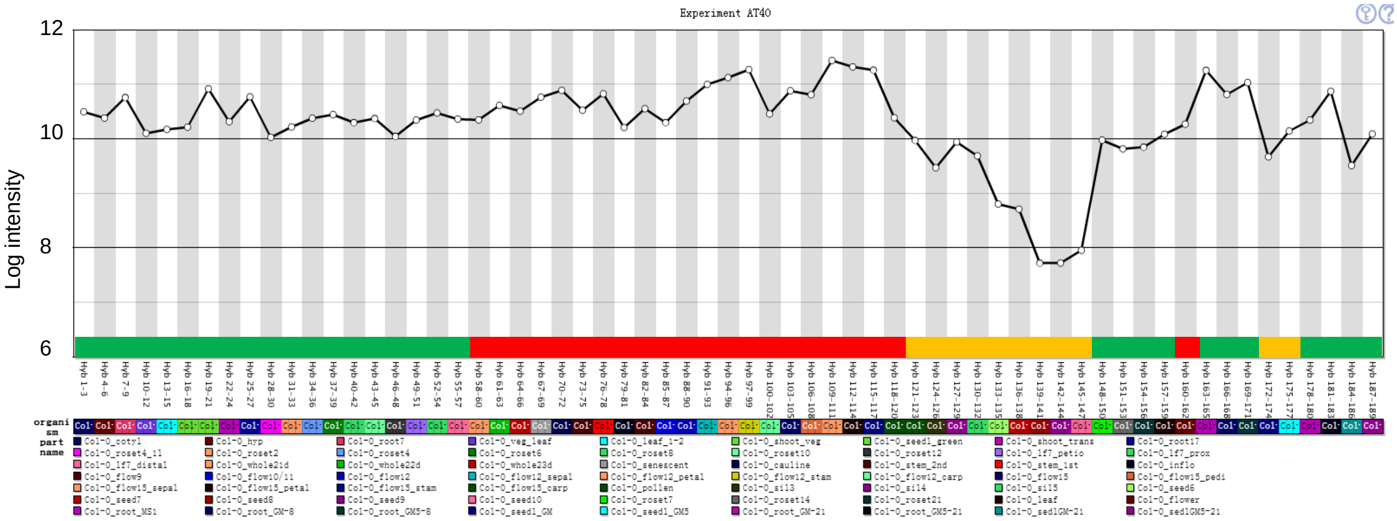

Figure S4A. Tissue-specific expression data of *AthMMT*s from PLEXdb.
The green, red, orange boxes represent vegetative tissues such as roots, stems and leaves, flowers, and seeds, respectively.

Figure S4 Zhao et al. 2018

GmaMMT1

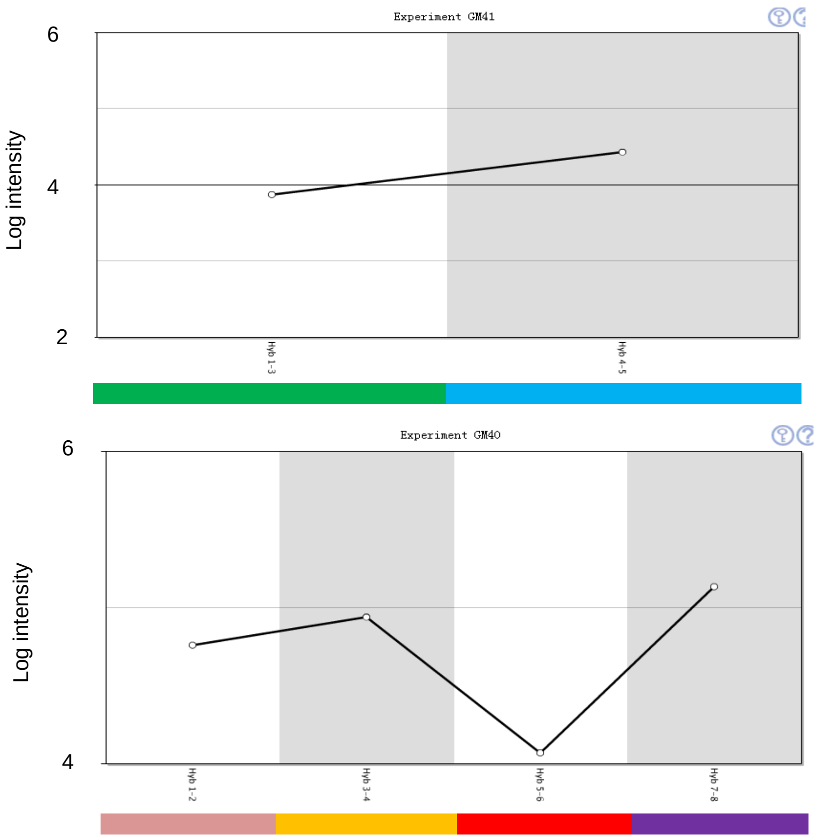

GmaMMT2

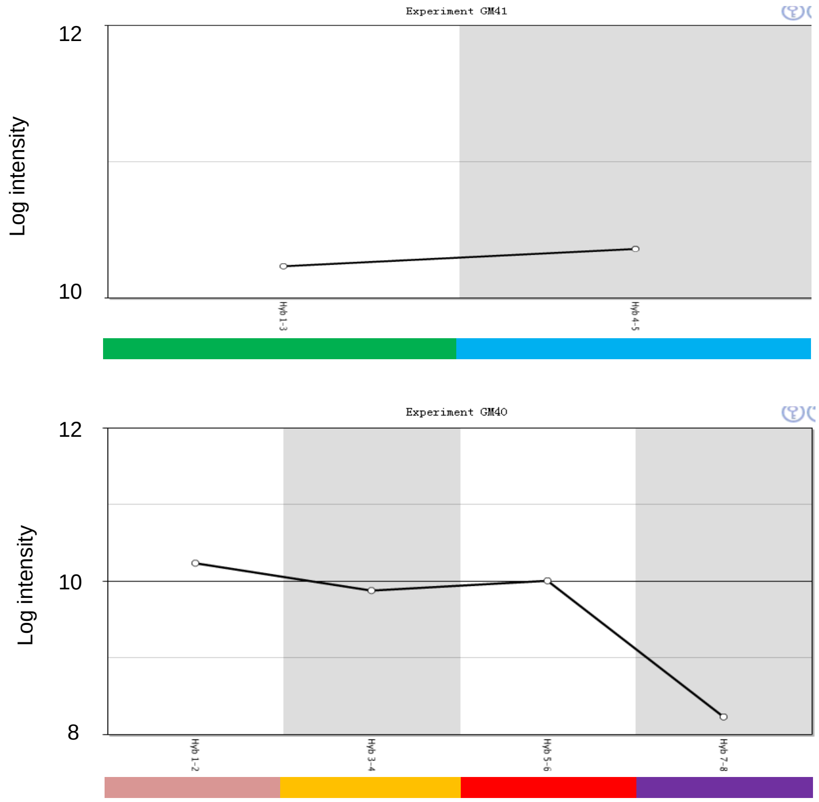

Figure S4B. Tissue-specific expression data of *GmaMMTs* from PLEXdb.
The green and blue boxes represent root apical meristem and leaf non-meristem. The pink, orange, red and purple boxes represent bean 2mm, bean 5mm, pod elongation and fully grown, respectively .
